# Nanotechnology‐Based Immunostimulants in Aquaculture: Mechanisms, Advances and Practical Applications

**DOI:** 10.1002/vms3.71158

**Published:** 2026-08-12

**Authors:** Mustafa Öz, Enes Üstüner

**Affiliations:** ^1^ Department of Fisheries and Diseases, Faculty of Veterinary Medicine Aksaray University Aksaray Turkey

**Keywords:** antimicrobial resistance, aquaculture disease management, controlled drug delivery, nano‐immunostimulants, polymeric nanocarriers

## Abstract

**Background:**

The rapid expansion of aquaculture has intensified disease outbreaks, increasing antibiotic reliance and accelerating antimicrobial resistance (AMR). Given the ecological and economic consequences of traditional treatments, nanotechnology‐based eco‐efficient alternatives are urgently needed.

**Objective:**

The primary goal of this manuscript is to critically evaluate the practical applicability, mechanisms of action and safety profiles of nano‐immunostimulants in aquaculture. Furthermore, this review aims to provide a comparative roadmap of major nano‐delivery systems (polymeric, metallic, lipid and carbon‐based), identifying their safety–efficacy trade‐offs to facilitate their transition from laboratory research to commercial aquaculture.

**Methods:**

A structured literature search was conducted across major databases (PubMed, Scopus, Web of Science and ScienceDirect) targeting nano‐immunostimulants, delivery systems, fish/shellfish immunity and environmental safety. Due to high methodological heterogeneity, data were synthesised qualitatively.

**Results:**

Polymeric nanocarriers (e.g., chitosan, alginate, PLGA) show the highest translational potential, significantly improving immune biomarkers, bioavailability and survival against major pathogens (e.g., *Aeromonas* and *Vibrio* spp.) with favourable safety profiles. While metallic, lipid and carbon‐based nanomaterials exhibit strong immunomodulatory and antimicrobial potential, their practical adoption is currently constrained by variable toxicity, environmental persistence and bioaccumulation risks.

**Conclusions:**

Nano‐immunostimulants represent a transformative strategy for antibiotic‐free, One Health‐oriented aquaculture. To achieve commercial viability, future research must shift from basic efficacy trials towards green synthesis, standardised ecotoxicological assessments and field‐scale cost‒benefit validation.

## Graphical abstract

Nano‐immunostimulants improve fish health through targeted and controlled delivery of immunostimulatory compounds. Biodegradable polymeric and lipid‐based nanocarriers enhance immune responses, bioavailability and disease resistance while reducing antibiotic dependence and environmental impact. These systems represent a promising sustainable strategy for precision aquaculture and One Health‐oriented disease management.

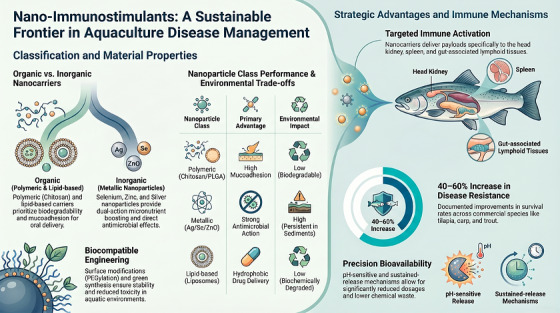

## 1 Introduction

The main production barriers in global aquaculture stem from three interconnected factors: recurrent disease outbreaks, feed and input waste, and environmental capacity limits in high‐intensity farming systems (Kaiser et al. 2020; Krkošek 2010). Disease outbreaks are promoted by dense host populations, weak biosecurity practices and environmental deterioration. Climate‐related stressors, including temperature fluctuations, salinity changes, acidification, severe weather and poor water quality, can directly impair fish immune competence by disrupting mucosal barriers, stress physiology, leukocyte activity and pathogen–host balance. As a result, environmentally stressed fish often become more susceptible to opportunistic bacterial, viral and parasitic infections, increasing the need for preventive health strategies that can support or ‘boost’ immune readiness without relying on antibiotics. These pressures increase production costs through mortality, reduced growth, treatment expenses and production instability (Gundi 2014; Matkarimov et al. 2025). Disease risks in aquaculture are highly species‐ and system‐dependent, reflecting host biology, life stage, stocking density, water quality and local pathogen ecology. Intensive finfish systems, including rainbow trout production, can favour bacterial outbreaks when high host density, environmental stress and pathogen exposure coincide (Maulu et al. 2024; Radosavljević et al. 2022). Parasitic infections remain important across many finfish and marine shellfish operations, while shrimp and crayfish are increasingly affected by emerging diseases associated with environmental stressors and production intensification (Hambali et al. 2024; L. Lu et al. 2025; Shields and Huchín‐Mian 2020). Seasonal shifts in marine microbial communities may further contribute to localised disease outbreaks, whereas freshwater species such as tilapia and snakehead differ in susceptibility according to species and life stage (Hambali et al. 2024; Matkarimov et al. 2025).

Although chemical treatments are still widely used to control parasitic and microbial threats, their repeated application can create ecological and regulatory concerns. Consequently, disease management increasingly requires integrated approaches that combine biosecurity, metagenomics/eDNA‐based surveillance, selective breeding, recirculating aquaculture systems, probiotics and other biotechnological tools to reduce species‐specific disease risks and production losses (Aly and Fathi 2024; Buchmann 2022; Prakoso et al. 2020; Tello et al. 2020). The application of antibiotics in aquaculture operations leads to the selection of antibiotic‐resistant bacteria (ARB) and the accumulation of antibiotic resistance genes (ARGs) throughout water bodies and sediments and host microbiomes, which accelerates antimicrobial resistance (AMR) development in aquatic ecosystems (Alotaibi 2023; Hambali et al. 2024; Yuan et al. 2023). Research shows that tetracycline and sulphonamide resistance genes remain active in pond and marine sediments after antibiotic treatment because environmental selection mechanisms work on these genes and mobile genetic elements that facilitate horizontal gene transfer between environmental bacteria and pathogenic bacteria (Muziasari et al. 2014; Muziasari et al. 2016; Tamminen et al. 2011). The process of antibiotic residue accumulation occurs in water environments and sediment deposits and cultured aquatic life, while aquaculture water and sediment contain ng/L to µg/g concentrations that directly affect ARG abundance and microbial community shifts, which demonstrate resistance transmission through bioaccumulation and ecological disruption (X. Lin et al. 2023; Xiong et al. 2015; Y. Zhang et al. 2023). The implementation of antibiotic use restrictions reduces selection pressure, but fish health management needs to adopt enhanced biosecurity practices and vaccination programs and non‐antibiotic prophylactic measures and metagenomics/eDNA surveillance for disease control according to current risk mitigation and One‐Health reviews (Lastauskienė et al. 2021; Zhao et al. 2025).

Disease risks in aquaculture are highly species‐ and system‐dependent, reflecting host biology, life stage, stocking density, water quality and local pathogen ecology. Intensive finfish systems, including rainbow trout production, can favour bacterial outbreaks when high host density, environmental stress and pathogen exposure coincide (Maulu et al. 2024; Radosavljević et al. 2022). Parasitic infections remain important across many finfish and marine shellfish operations, whereas shrimp and crayfish are increasingly affected by emerging diseases associated with environmental stressors and production intensification (Hambali et al. 2024; L. Lu et al. 2025; Shields and Huchín‐Mian 2020). Although chemical treatments are still widely used, repeated application raises ecological and regulatory concerns. Therefore, disease management increasingly requires integrated approaches combining biosecurity, eDNA/metagenomic surveillance, selective breeding, recirculating aquaculture systems, probiotics and other biotechnological tools to reduce species‐specific disease risks and production losses (Aly and Fathi 2024; Buchmann 2022; Prakoso et al. 2020; Tello et al. 2020).

Nanoparticles (NPs) possess distinctive characteristics, including a high surface area‐to‐volume ratio and adjustable size, charge and surface modification capabilities that result in better payload protection, controlled release and targeted tissue delivery than traditional carriers (Pachar et al. 2024). The features enhance both nutrient and immunostimulant solubility and cell penetration, which results in better bioavailability and reduced medication needs (Pachar et al. 2024). The protective methods of nanoencapsulation, such as liposomes, chitosan NPs and nanoemulsions, prevent water degradation and digestive elimination of sensitive compounds while delivering them through controlled release at mucosal and systemic sites, as demonstrated by fish studies using liposome‐encapsulated LPS/dsRNA and basil oil nanoemulsions (El‐Ekiaby 2019; Ruyra et al. 2013). At the nanoscale, delivery systems can enhance uptake by macrophages and other antigen‐presenting cells (APCs), thereby supporting innate immune responses such as cytokine release and respiratory burst activity (Cui et al. 2005; Ruyra et al. 2013). Compared with conventional bulk formulations, these systems may reduce the required doses, improve delivery to gill, intestinal, lymphoid and phagocytic tissues, and prolong immunomodulatory activity through controlled release and adjuvant‐like effects (Cui et al. 2005; Ruyra et al. 2013).

This structured narrative review addresses the need for a critical synthesis of nano‐immunostimulant (NIS) safety, efficacy, mechanisms and practical applicability in aquaculture. Rather than presenting a formal systematic review or meta‐analysis, it integrates evidence from experimental, mechanistic, toxicological and applied studies to compare major NP classes across aquaculture species. The review focuses on four main questions: which nano‐delivery systems show the most favourable safety–efficacy balance; how NP properties influence immune activation, bioavailability and disease resistance; what risks are associated with toxicity, bioaccumulation, environmental persistence and non‐target exposure; and which regulatory, economic and scalability barriers must be addressed before commercial implementation.

### 1.1 Literature Search Strategy and Review Framework

To ensure transparency and comprehensiveness, this manuscript follows a structured narrative review methodology rather than a rigid systematic review or meta‐analysis. A rigourous and reproducible literature search was executed across five major electronic databases: PubMed, Scopus, Web of Science, ScienceDirect and Google Scholar. The search covered literature published from database inception up to January 2026.

The search strategy relied on specific Boolean operators combining primary and secondary keywords: (‘nano‐immunostimulant’ OR ‘nanoparticle’ OR ‘nanocarrier’ OR ‘nanoemulsion’ OR ‘liposome’) AND (‘aquaculture’ OR ‘fish immunity’ OR ‘shellfish immunity’ OR ‘shrimp’ OR ‘trout’ OR ‘tilapia’ OR ‘carp’) AND (‘disease resistance’ OR ‘antimicrobial resistance’ OR ‘toxicity’ OR ‘bioaccumulation’ OR ‘environmental risk’).

To establish a well‐defined boundary for study selection, explicit inclusion and exclusion criteria were implemented:

- *Inclusion criteria*: (1) Peer‐reviewed primary experimental studies or recent high‐impact mechanistic reviews; (2) research evaluating engineered NPs (polymeric, metallic, lipid‐based or carbon‐based) specifically applied as immunostimulants, adjuvants or vaccine delivery vehicles; and (3) studies reporting clear immunological, haematological, antioxidant, histopathological or pathogen challenge outcomes in economically important aquaculture finfish or crustacean species.
- *Exclusion criteria*: (1) Studies focusing exclusively on non‐aquatic biomedical applications or human medicine; (2) articles evaluating bulk/macro‐scale compounds without nanoscale characterisation; (3) dynamic environmental monitoring papers that did not explicitly assess fish or shellfish health outcomes; (4) dissertations, conference abstracts and non‐peer‐reviewed white papers.

The article screening process was carried out in three distinct phases: (1) Initial screening of titles and abstracts to eliminate duplicates and clearly irrelevant records; (2) full‐text evaluation of remaining articles against the predefined inclusion/exclusion criteria; and (3) a qualitative synthesis of the finalised literature. Due to high methodological heterogeneity across host species, NP types, dosing regimens and pathogen challenge models, a formal quantitative meta‐analysis was not mathematically feasible or intended. Instead, data were synthesised qualitatively to map overarching mechanisms, translational trade‐offs and environmental safety profiles.

## 2 Nanotechnology Applications in Aquaculture: An Overview

### 2.1 Fundamentals of Aquaculture Nanotechnology

NPs are useful in fish health applications because of their small size, high surface‐area‐to‐volume ratio and tuneable surface chemistry. These properties can improve mucosal and cellular uptake, protect labile bioactive compounds and enable controlled delivery at lower doses than conventional bulk formulations (Pachar et al. 2024; Ruyra et al. 2013). In aquatic systems, NP behaviour is governed by diffusion, Brownian motion, colloidal stability, zeta potential and electrostatic or steric stabilisation. Persistence and biological interactions are further influenced by aggregation kinetics, ionic strength, dissolved organic matter (DOM), surface reactivity, ion release and reactive oxygen species generation (Nguyen et al. 2024; S. Zhang, Lu, et al. 2022). In aquaculture systems, NP stability is affected by salinity, pH, chloride concentration, organic matter, biofilm formation and protease activity, particularly for silver NPs (AgNPs). Formulation strategies such as chitosan or alginate capping, surface modification and particle‐size control can help maintain biological activity while reducing toxicity under farm‐water conditions (Luis et al. 2021; S. Zhang, Lu, et al. 2022). These principles highlight the need for rational NP design before laboratory findings can be translated into safe and effective aquaculture applications (Phanse et al. 2022; Ruyra et al. 2013).

### 2.2 Selection Criteria for Fish Immunostimulation

Based on the physicochemical principles described above, NP selection for fish immunostimulation should consider particle size, surface charge, colloidal stability, biodegradability, payload protection, immune targeting and environmental safety.

Key criteria for selecting NPs for fish immunostimulation include the following:

- *Particle size, surface charge and colloidal stability*: These properties determine mucosal absorption, epithelial transport, immune cell uptake and payload retention. Positively charged chitosan‐based NPs, for example, can improve antigen absorption and intestinal residence through mucoadhesive interactions (Guadarrama‐Escobar et al. 2023; Ji et al. 2015; Y. Liu et al. 2021). For oral aquaculture applications, colloidal stability should also be assessed under field‐relevant pond water temperatures rather than only under constant laboratory conditions. Temperature fluctuations can influence NP size distribution, zeta potential, viscosity, aggregation behaviour and payload‐release kinetics, particularly in mucoadhesive systems such as chitosan NPs. Warmer pond conditions may accelerate polymer relaxation, diffusion and release, whereas colder conditions may reduce diffusion and alter NP–mucus interactions. Therefore, candidate NISs should be screened across realistic temperature ranges that reflect the target species and farming environment to ensure stable feed incorporation, water‐exposure tolerance and predictable mucosal delivery.
- *Biocompatibility and biodegradability*: Feed‐delivered NPs should have low toxicity, predictable degradation and minimal residue formation. Chitosan‐ and alginate‐based systems are frequently favoured because they are biodegradable and generally compatible with oral administration in fish (Gao et al. 2016; Madrid et al. 2021; Rosidah and Mulyani 2022).
- *Payload protection and gut release*: Nanocarriers should protect labile antigens or immunostimulants from gastric degradation while enabling release near gut‐associated immune tissues. Chitosan coatings and alginate layers are commonly used for this purpose (Fitriagustiani et al. 2022; Gao et al. 2016).
- The co‐delivery of adjuvants with nucleic acids occurs through formulations that merge peptides with DNA and poly(I:C) and PADRE immunostimulants to produce enhanced synergistic effects (Correia‐Pinto et al. 2015; Ramos et al. 2005).

How NP classification affects digestive interactions and absorption

- Polymeric/organic NPs made from chitosan and alginate function as mucoadhesive agents to improve permeation and form protective gels in acidic stomach conditions, which lengthen their GI tract residence time for better antigen presentation to stomach and intestinal epithelial cells (Alagarsamy et al. 2024; Y. Liu et al. 2021). The drug delivery system becomes more stable, and its absorption through mucosal tissues improves when using chitosan derivatives that include thiolated and amphiphilic modifications (Uchegbu et al. 2014; Werle and Bernkop‐Schnürch 2008).
- The delivery system of lipid‐based nanoemulsions functions as a mucosal adhesion enhancer to protect vaccine contents on mucosal surfaces before they reach immune tissues located in mucosal areas (Lusiastuti et al. 2025).
- The stability of inorganic NPs, including silica, maintains their integrity in harsh gastrointestinal conditions, but their restricted dimensions and digestive process‐induced aggregation or dispersion prevent them from passing through the intestinal epithelial barrier (Lusiastuti et al. 2025; Ribeiro et al. 2023).

Comparative advantages and limitations: organic versus inorganic NPs in feeds

- Organic NPs, particularly polymeric and lipid‐based systems, offer biodegradability, low toxicity, mucoadhesion, payload protection, sustained release and co‐delivery of adjuvants or nucleic acids. These advantages make them suitable for oral antigen or immunostimulant delivery in fish. Their main limitations are reduced stability under harsh gastrointestinal or water conditions and batch‐to‐batch variability during scale‐up (Correia‐Pinto et al. 2015; Fitriagustiani et al. 2022; Gao et al. 2016).
- Inorganic NPs provide high physical and chemical stability, tuneable surface chemistry and strong antimicrobial or immunomodulatory activity. However, their use in feed applications is limited by potential aggregation during digestion, restricted epithelial passage, metal ion release, oxidative stress, tissue accumulation and environmental persistence. Therefore, inorganic systems require stricter dose optimisation and toxicological validation before commercial use (Handy et al. 2008; Lusiastuti et al. 2025; Ribeiro et al. 2023).

**FIGURE 1:**
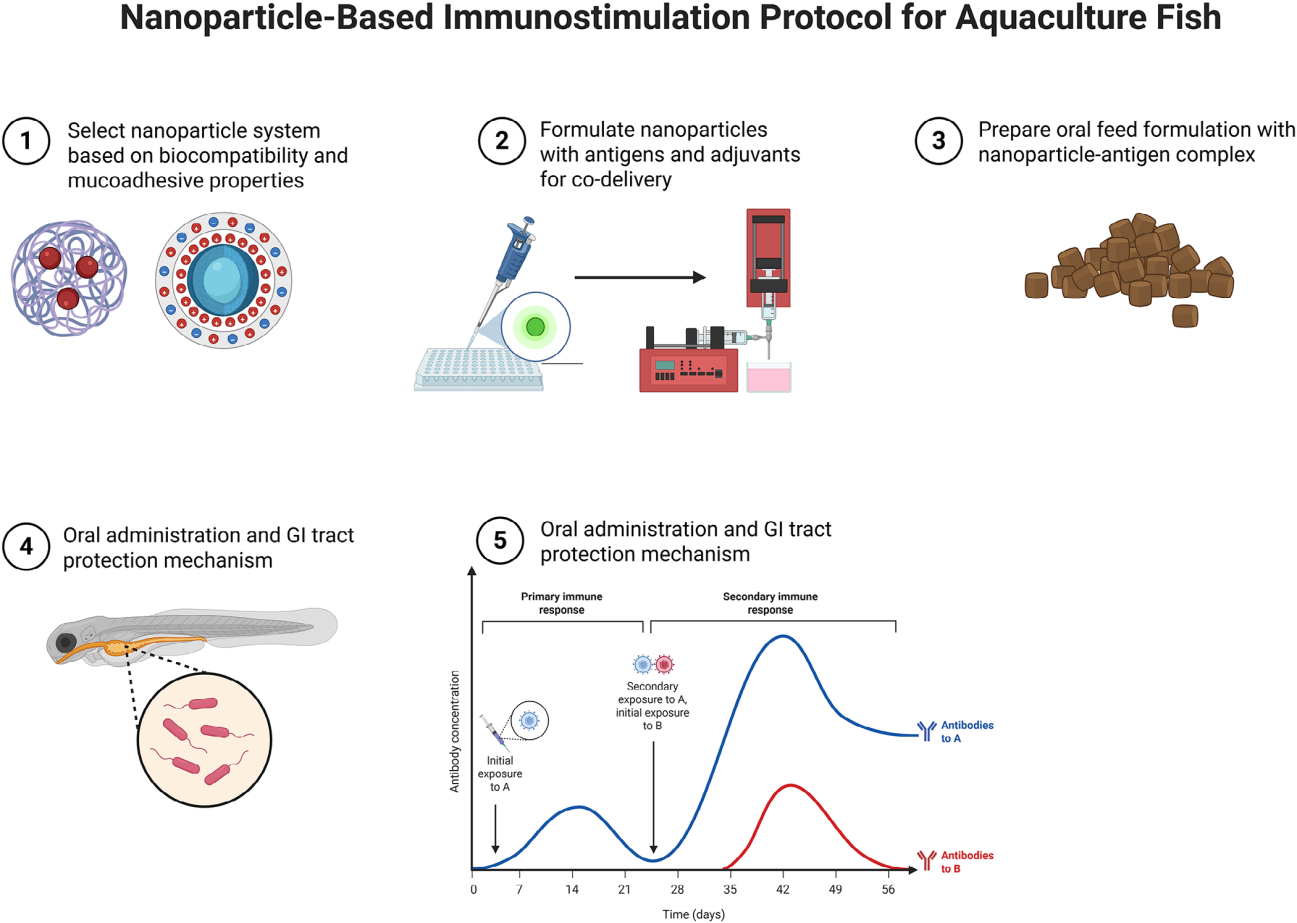
Conceptual illustration of the operational workflow for nanoparticle‐based immunostimulation in aquaculture, outlining stages from particle selection to biological evaluation.

The research findings show that GIT antigen protection and mucoadhesive permeation enhancement should be the main focus of selection for chitosan‐based organic systems that need to stay stable and biodegradable, but inorganic NPs help with these goals while creating safety issues and aggregation problems (Gao et al. 2016; Ji et al. 2015; Lusiastuti et al. 2025). A conceptual workflow for NP‐based immunostimulation in aquaculture fish, from particle selection to immune response evaluation, is illustrated in Figure 1.

### 2.3 Classification of NPs by Material

#### 2.3.1 Metallic NPs

Among the various NP types meeting the selection criteria described above, metallic NPs—particularly selenium, zinc and silver—have received considerable attention for fish immunostimulation.

**FIGURE 2:**
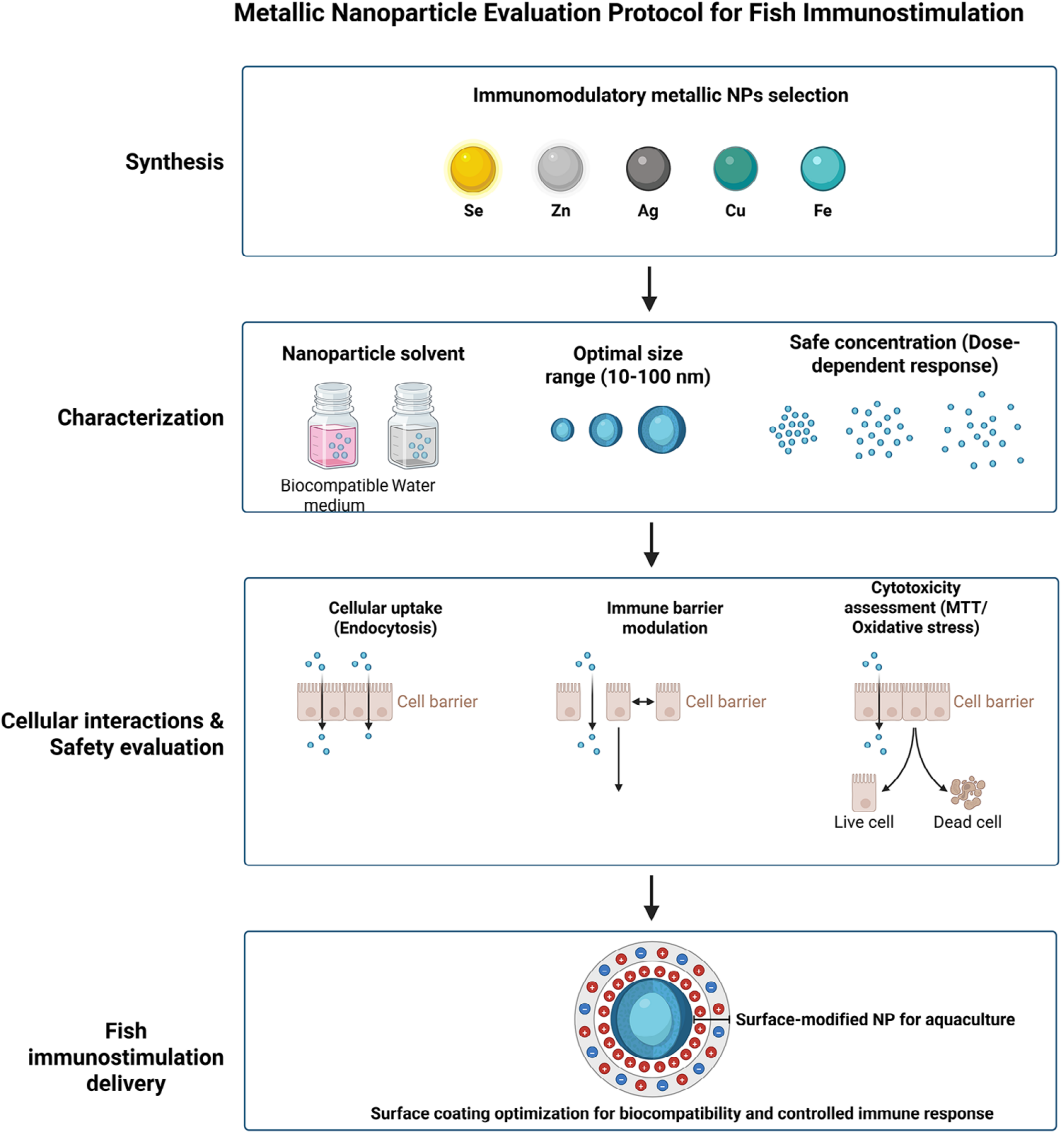
Conceptual framework for evaluating metallic nanoparticles in fish health, highlighting parameters for physicochemical profiling and safety screening.

Selenium NPs (SeNPs), zinc NPs and AgNPs can modulate fish immunity through partly distinct but overlapping mechanisms. SeNPs may enhance growth, antioxidant defence and disease resistance when supplied at appropriate doses, but excessive exposure can be toxic (Dezfouli et al. 2019; Saad et al. 2022). Zinc oxide NPs (ZnONPs) and other zinc‐based NPs can alter immune gene expression and inflammatory pathways; however, their effects depend strongly on concentration, surface modification and developmental stage (Choi et al. 2016; Kaur et al. 2019). AgNPs show antimicrobial and immunomodulatory activity, but responses are species‐, dose‐, size‐ and surface‐dependent, and toxicity has been reported in fish cell lines and embryos (George et al. 2012; Shaalan et al. 2017). For metallic NPs, particle size, surface coating, dissolution rate and exposure route are major determinants of both efficacy and toxicity. Although particles in the 10–100 nm range are often considered favourable for cellular uptake, this range should not be interpreted as universally safe. Biocompatible coatings may improve dispersion and reduce uncontrolled cellular entry, but risk assessment must also consider metal ion release, oxidative stress, immune signalling disruption and tissue accumulation (Długosz et al. 2020; Handy et al. 2008; Ngobili and Daniele 2016). Thus, metallic NPs remain promising but require stricter dose optimisation and safety validation than biodegradable polymeric carriers. A conceptual evaluation workflow for metallic NPs in fish immunostimulation applications is presented in Figure 2.

#### 2.3.2 Polymeric NPs

In contrast to metallic NPs, biodegradable polymeric carriers offer distinct advantages in terms of controlled degradation and reduced environmental persistence.

Compared with many metallic systems, biodegradable polymeric NPs generally offer a more favourable safety profile for feed‐based aquaculture applications. Chitosan, alginate and PLGA‐like carriers can provide controlled degradation, payload protection and sustained antigen or immunostimulant release while reducing concerns related to metal ion release and long‐term environmental persistence (Dixit et al. 2014; Nicolete et al. 2011; Verma et al. 2018). However, polymeric systems are not risk‐free; their performance depends on polymer composition, molecular weight, particle size, surface modification, encapsulation efficiency and stability in feed and gastrointestinal conditions. Polymeric carriers enable extended targeted delivery of substances while functioning as self‐adjuvanting systems that control both mucosal and systemic immune responses without releasing dangerous metal substances (Dixit et al. 2014; Z. Yu et al. 2023). Chitosan‐ and PLGA‐based particles differ in release behaviour and immune interaction. Chitosan is pH‐responsive and mucoadhesive, which may improve intestinal retention, mucosal uptake and local immune activation (Chua et al. 2011; Dawood et al. 2020). PLGA‐based systems can provide diffusion‐controlled antigen release and efficient uptake by APCs, supporting systemic antigen presentation (Nicolete et al. 2011; Vivek et al. 2014). For oral fish applications, optimal polymers should combine biodegradability, mucoadhesion, suitable molecular weight, stable encapsulation and appropriate surface modification to balance residence time, uptake and immune activation (Chua et al. 2011; Palmiero et al. 2018; Tang et al. 2009).

#### 2.3.3 Lipid‐Based Nanocarriers

Lipid‐based systems provide an alternative approach for delivering hydrophobic immunostimulants while maintaining biocompatibility.

Lipid‐based nanocarriers are particularly useful for hydrophobic immunostimulants that have poor solubility or limited stability in conventional formulations. By embedding active compounds in lipid matrices, these systems can protect payloads from degradation, promote sustained release and improve intestinal absorption and mucosal contact (Ji et al. 2015; Lehn et al. 2014). Depending on their composition and surface properties, lipid NPs may also cross mucus barriers and enter enterocytes, thereby enhancing the systemic absorption of hydrophobic compounds (Hogarth et al. 2021; Ji et al. 2015). However, lipid systems may be unstable in aquaculture environments because dilution, temperature shifts, salinity, microbial activity and surfactant depletion can promote Ostwald ripening, coalescence, or phase separation. Solid lipid NPs, stabilised nanoemulsions, optimised surfactants and tailored lipid combinations can partly address these limitations (Hogarth et al. 2021; Lehn et al. 2014). Vesicle composition also affects membrane fluidity, permeability, protein corona formation, endocytic uptake, lymphatic transport and tissue distribution to the gut, liver and spleen (S. Ahmed et al. 2011). Therefore, lipid‐based systems require formulation‐specific optimisation for payload compatibility, storage stability and immune targeting.

#### 2.3.4 Carbon‐Based Nanomaterials

Carbon‐based nanomaterials are emerging candidates for immunostimulant, adjuvant and vaccine delivery applications, but their use in aquaculture remains limited by safety concerns. Functionalised carbon nanotubes (CNTs) and graphene‐based materials may interact with macrophages and APCs through membrane contact and endocytic uptake pathways. However, pristine or poorly functionalised forms can form intracellular aggregates and may induce cytotoxicity, inflammation, or asbestos‐like pathogenicity, indicating the need for safety‐by‐design approaches before aquaculture use (Y. Liu et al. 2013; Orecchioni et al. 2014; S. P. B. Sousa et al. 2020). Their biodistribution and immunomodulatory effects in the gills, gut, liver and spleen depend on surface chemistry, functionalisation, particle size, uptake route and membrane interactions (Marchesan et al. 2015; Orecchioni et al. 2014; S. P. B. Sousa et al. 2020). CNTs may also interact with organic pollutants and influence their persistence, transport and bioavailability in aquatic systems, which complicates environmental risk assessment (Petersen et al. 2011; S. P. B. Sousa et al. 2020; Su et al. 2013). Fish immunostimulation benefits need thorough hazard evaluation and controlled functionalisation and environmental impact assessment to achieve safe and effective applications (Orecchioni et al. 2014; Petersen et al. 2011; S. P. B. Sousa et al. 2020). The physicochemical properties and functional trade‐offs of major NP classes, including polymeric, metallic and lipid‐based systems, are synthesised in Table 1. Overall, the available evidence suggests that no single NP class is optimal for all aquaculture applications. Polymeric systems, especially chitosan‐, alginate‐ and PLGA‐based carriers, appear most suitable for oral immunostimulant and vaccine delivery because they combine biodegradability, mucosal adhesion, payload protection and relatively favourable safety profiles. However, their performance depends strongly on formulation stability, polymer source, molecular weight, encapsulation efficiency and feed‐processing conditions. Metallic and metal oxide NPs often show stronger antimicrobial or trace element‐related immunomodulatory effects, but their narrower safety margin, oxidative stress potential, tissue accumulation and environmental persistence limit their direct translation to routine feed use. Lipid‐based systems are promising for hydrophobic immunostimulants and mucosal delivery, yet storage stability, oxidation, surfactant selection and performance in variable salinity conditions remain important barriers. Carbon‐based nanomaterials have high loading capacity and surface functionality, but their safety database in aquaculture is still insufficient. Therefore, current evidence favours biodegradable polymeric and selected lipid‐based systems for near‐term application, whereas metallic and carbon‐based materials require more extensive toxicological and environmental validation before broad commercial adoption.Table 1 summarises the comparative features of organic and inorganic NP delivery systems in aquaculture, including delivery mechanisms, advantages, limitations, relative production‐cost considerations and environmental stability.

**TABLE 1:** Comparative analysis of organic and inorganic nanoparticle delivery systems in aquaculture.

Nanoparticle class	Delivery mechanism	Advantages	Limitations	Environmental stability	Estimated production cost
*Polymeric nanoparticles* (e.g., chitosan, PLGA‐like carriers, quercetin or thymol in nanoform, nanospheres)	Typically, *oral via feed* as encapsulated nutraceuticals, immunostimulants or drug/vaccine carriers; uptake mainly through *intestinal endocytosis* and transcytosis; can be engineered for controlled release and targeting	Generally, *biodegradable and biocompatible*, improving *drug/nutrient solubility, protection and bioavailability*; can enhance growth, feed utilisation, antioxidant status and immunity; allow controlled or sustained release and reduced dosing frequency	Less standardised than metal NPs; synthesis and scale‐up can be more complex; stability in feed and water can vary; toxicity usually low but still not fully characterised for all polymers and surface chemistries	Tend to be less persistent and more biodegradable than inorganic NPs; environmental fate depends on polymer type, but generally lower long‐term accumulation and ecotoxicity concerns than metals or carbon‐based materials	Low to medium. Natural polymers such as chitosan and alginate are generally inexpensive, scalable and compatible with feed‐based delivery, whereas PLGA‐like carriers, functionalised polymers and highly controlled nano‐formulations may increase production and quality‐control costs
	El‐Naby et al. (2020); D. Ibrahim et al. (2021); Ismael et al. (2021); Moniruzzaman and Min (2020)	El‐Naby et al. (2020); Abdel‐Latif et al. (2021); D. Ibrahim et al. (2021); Ismael et al. (2021)	Batir‐Marin et al. (2025); Moniruzzaman and Min (2020)	Batir‐Marin et al. (2025); Moniruzzaman and Min (2020); Shaw and Handy (2011)	
*Metallic and metal–oxide nanoparticles* (e.g., Ag, Cu, ZnO, Se, TiO_2_, Fe_2_O_3_)	*Waterborne exposure* (disinfection, pond treatment) via gills/skin; *oral via feed* as micronutrient or antimicrobial supplements (ZnO, Se, Fe, Cu); act directly on pathogens in water or gut; uptake via gills, gut epithelium and sometimes skin	Strong antimicrobial and immunomodulatory actions (Ag, Cu, ZnO); trace‐element forms (Se, Zn, Cu, Fe) can improve growth, antioxidant defences, immune status and stress resistance at optimised doses	Narrow margin between beneficial and toxic doses; can *induce oxidative stress, organ damage, behavioural changes and reproductive disruption* at sublethal levels; risk of *bioaccumulation and food‐chain transfer*; toxicity often differs from ionic forms and is species‐ and size‐dependent	Often *highly persistent* in water and sediments; can aggregate but still bioavailable; metal oxides and Ag/CuNPs can bioaccumulate in fish tissues and pose *long‐term ecotoxicological and human‐health risks*, although dissolution and speciation strongly modulate persistence and hazard	Medium to high. Raw material costs vary by metal type, but controlled synthesis, coating or stabilisation, particle‐size control, purification, waste management and stricter toxicological validation can increase overall production and regulatory costs
	Çiçek and Özoğul (2021); Dube (2024); Malhotra et al. (2020); Mohtashemipour et al. (2024); Shaalan et al. (2017); Shaw and Handy (2011); Yaqub et al. (2023)	Çiçek and Özoğul (2021); Dube (2024); Malhotra et al. (2020); Mohtashemipour et al. (2024); Yaqub et al. (2023)	Canli et al. (2018); Ostaszewska et al. (2018); Parsai and Kumar (2020); Pitt et al. (2018); Rahman et al. (2022)	Canli et al. (2018); Griffitt et al. (2009); Malhotra et al. (2020); Ostaszewska et al. (2018); Parsai and Kumar (2020); Pitt et al. (2018); Shaw and Handy (2011)	
*Lipid‐based nanoparticles* (e.g., nanoemulsions, liposomes, lipid nanospheres)	Mainly *oral via feed* or *immersion/vaccination* as carriers for lipophilic drugs, vaccines, essential oils or nutraceuticals; enter via *endocytosis and membrane fusion*, enhancing uptake across gut and mucosal barriers	High *biocompatibility and flexibility*; excellent for *hydrophobic drug and vaccine delivery*, improving stability, solubility, targeting and adjuvant effect; can enhance immune responses and reduce required doses of bioactives; easily combined with natural compounds (curcumin, essential oils)	More physically fragile than solid NPs (oxidation, coalescence); production and cold‐chain requirements can be costlier; data on chronic toxicity and residues in aquaculture systems are still limited compared with metals	Composed of *lipids that are readily degraded* biochemically; generally *low long‐term persistence*, although surface stabilisers and encapsulated actives can alter fate; likely lower ecotoxicity than metals or durable carbon nanomaterials but systematic field data are scarce	Medium. Production cost depends on lipid source, surfactant or stabiliser quality, encapsulation method, oxidation control, storage conditions and cold‐chain or stability requirements, especially for liposomes and nanoemulsions
	Batir‐Marin et al. (2025); Dube (2024); Moniruzzaman and Min (2020); Subaramaniyam et al. (2023)	Batir‐Marin et al. (2025); Dube (2024); Moniruzzaman and Min (2020); Subaramaniyam et al. (2023)	Batir‐Marin et al. (2025); Moniruzzaman and Min (2020)	Batir‐Marin et al. (2025); Dar et al. (2020)	
*Carbon‐based nanoparticles/nanoplastics* (e.g., polystyrene nanoplastics, C_60_ fullerenes, CNTs, graphene‐like materials)	Predominantly *waterborne exposure*; taken up via ingestion (gut) and, for smaller sizes, via *chorion and epidermis* in embryos and larvae; distribute to multiple organs (gut, liver, brain, pancreas, eyes) and can translocate within developing fish	Some engineered forms (e.g., carbon nanotubes, graphene) have potential as *biosensors, delivery platforms or adsorbents*, but most current relevance in fish is as pollutants rather than deliberate health tools	Often *toxic at environmentally relevant ranges*: induce oxidative stress, behavioural alterations, cardiophysiological effects, growth retardation, immune perturbation, embryotoxicity and enhanced susceptibility to pathogens; can act as vectors for metals or other pollutants; limited demonstrated direct ‘health‐promoting’ uses in fish to date	Generally, *highly persistent* and prone to *bioaccumulation*; polystyrene and other nanoplastics can accumulate in multiple organs and show slow depuration; fullerenes (C_60_) similarly show persistence and chronic toxicity concerns in aquatic systems	High. Engineered carbon nanomaterials often require costly synthesis, functionalisation, purification, dispersion control, characterisation and safety validation; nanoplastics are mainly relevant as pollutants rather than intentional aquaculture inputs
	Mattsson et al. (2015); Pitt et al. (2018); Sendra et al. (2021); Sielska and Skuza (2025); Van Pomeren et al. (2017)	Moniruzzaman and Min (2020); Sendra et al. (2021); Sielska and Skuza (2025); Subaramaniyam et al. (2023)	Mattsson et al. (2015); Pitt et al. (2018); Sendra et al. (2021); Sielska and Skuza (2025); Van Pomeren et al. (2017)	Mattsson et al. (2015); Pitt et al. (2018); Sendra et al. (2021); Sielska and Skuza (2025); Van Pomeren et al. (2017)	

### 2.4 Nano‐Formulation Strategies for Targeted Delivery

Targeted nano‐formulation strategies should link particle design to fish immune anatomy, administration route, environmental exposure and safety constraints. Key design priorities include immune cell targeting, biomimetic surface engineering, material selection, biodistribution control and environmental risk minimisation.

*Immune‐cell and tissue targeting*: Ligand‐functionalised or biomimetic NPs can be designed to interact with macrophage‐ and dendritic‐like cell receptors and to improve delivery to the spleen, head kidney, gills and gut‐associated lymphoid tissues (Irvine et al. 2015; Ji et al. 2015; Uribe et al. 2011).

*Biomimetic surface design*: Natural membrane components, receptor‐specific ligands, or immune cell‐mimicking coatings may enhance selective uptake by immune cells and reduce premature clearance, as suggested by biomimetic targeting and vaccine delivery principles (Ji et al. 2015; Luk et al. 2018).

*Material selection*: Materials such as chitosan, alginate, PLGA and selected metal oxides should be chosen according to biodegradability, immunomodulatory activity, dose‐dependent toxicity and environmental persistence. Even materials with immunostimulatory potential require careful immunotoxicological evaluation (Dawood et al. 2020; He et al. 2022; Perera and Pathiratne 2014).

*Biodistribution control*: Particle size, surface charge, aggregation state and protein corona formation should be optimised to improve formulation stability, mucosal transport, immune‐organ delivery and clearance behaviour in fish (J. Chen et al. 2022; Gökşen et al. 2024; Kashiwada 2006).

*Environmental safety*: Formulation design should anticipate release into water and sediments, exposure of non‐target organisms, and possible food chain transfer. Environmental fate and ecotoxicity assessment should therefore be incorporated into early‐stage NIS development (Anne‐Catherine et al. 2016; Cedervall et al. 2012; Mattsson et al. 2015).

### 2.5 Synthesis Methods and Characterisation Techniques

**FIGURE 3:**
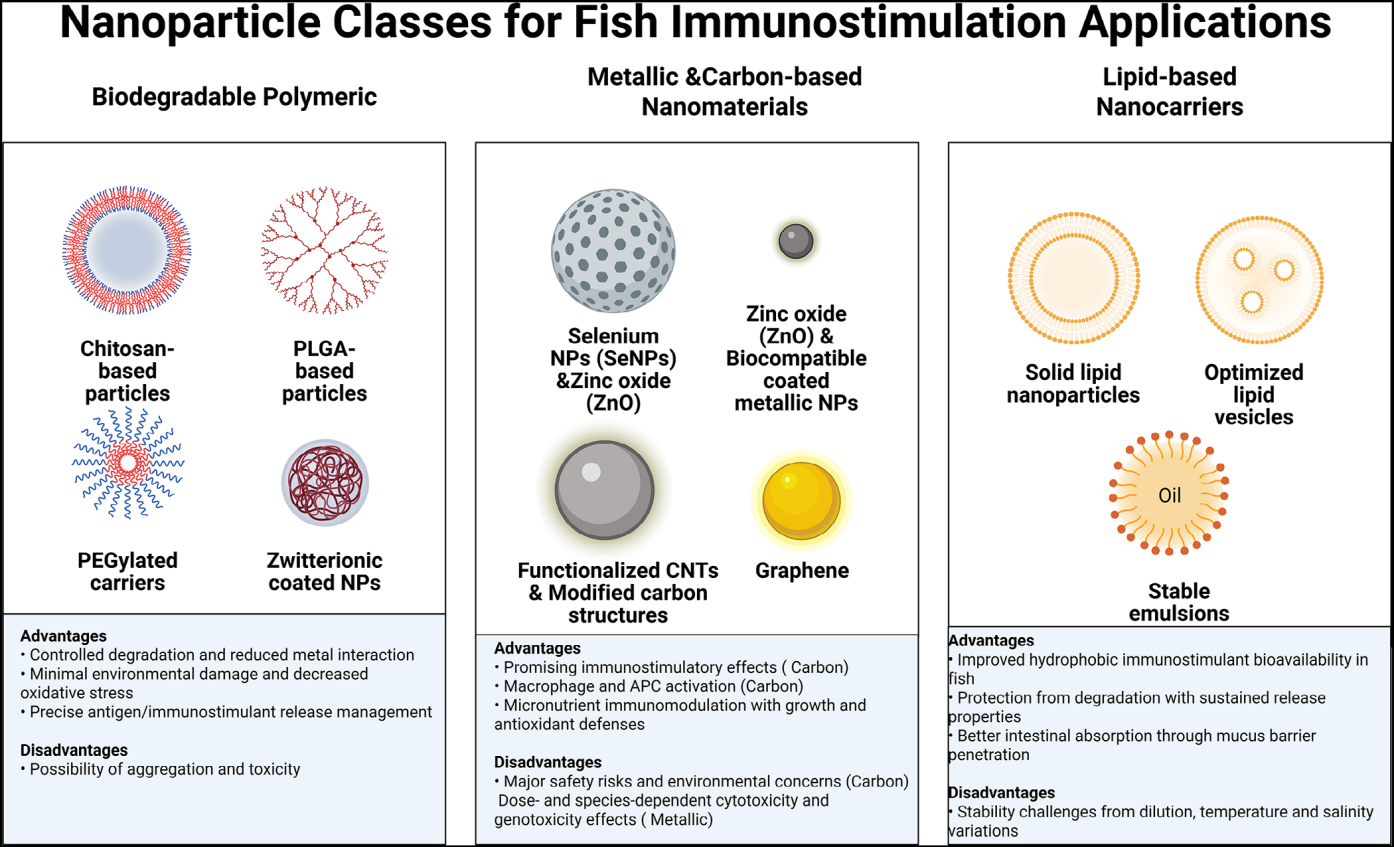
Evidence‐based classification framework comparing the immunomodulatory potential, delivery functions and primary safety boundaries of major nanomaterial classes.

NP synthesis determines physicochemical properties that directly influence safety, efficacy, reproducibility and environmental behaviour in fish systems. Chemical synthesis allows rapid and controlled production, but residual reagents and by‐products must be removed to avoid toxicity or immune disruption (Rajkumar et al. 2015). Biological or green synthesis can generate biocompatible surfaces with lower toxicity and good colloidal stability, provided that reaction media, biological extracts and process conditions are standardised (Jayachandran et al. 2021; Sana et al. 2020). Physical methods offer solvent‐free alternatives, but they may require high energy input and produce broad particle‐size distributions, complicating reproducibility and safety assessment (Domiszewski and Mierzejewska 2021; Modena et al. 2019). Product quality and safety assessments should include particle‐size distribution, zeta potential, morphology, crystallinity, agglomeration state, dissolution behaviour and protein corona formation under relevant physiological and aquaculture conditions. These parameters influence colloidal stability, biological uptake, immune activation and environmental fate (Domiszewski and Mierzejewska 2021; Modena et al. 2019). Fish exposure studies should also report haematological, biochemical, oxidative stress, immune and histopathological endpoints, including RBC, WBC, haemoglobin, haematocrit, liver and kidney biomarkers, antioxidant enzymes, cytokine expression and tissue lesions where applicable (Aluko et al. 2020; Dawood et al. 2020; Muthuswami and Mekala 2023; Rajan and Sakthiprabha 2022; Shaluei et al. 2013). Scaling green synthesis requires standardised biological inputs, controlled particle‐size production, reproducible surface modification and real‐time analytical monitoring to meet safety and regulatory expectations (Grainger and Castner 2008; Jayachandran et al. 2021; Sana et al. 2020). In addition, dissolution, bioavailability and environmental behaviour should be modelled under realistic aquaculture conditions before large‐scale implementation (Griffitt et al. 2007). The major NP systems used for fish immunostimulation, together with their advantages and limitations, are summarised in Figure 3.

## 3 Mechanisms of Action of NISs

### 3.1 Fish Immune System Architecture

**FIGURE 4:**
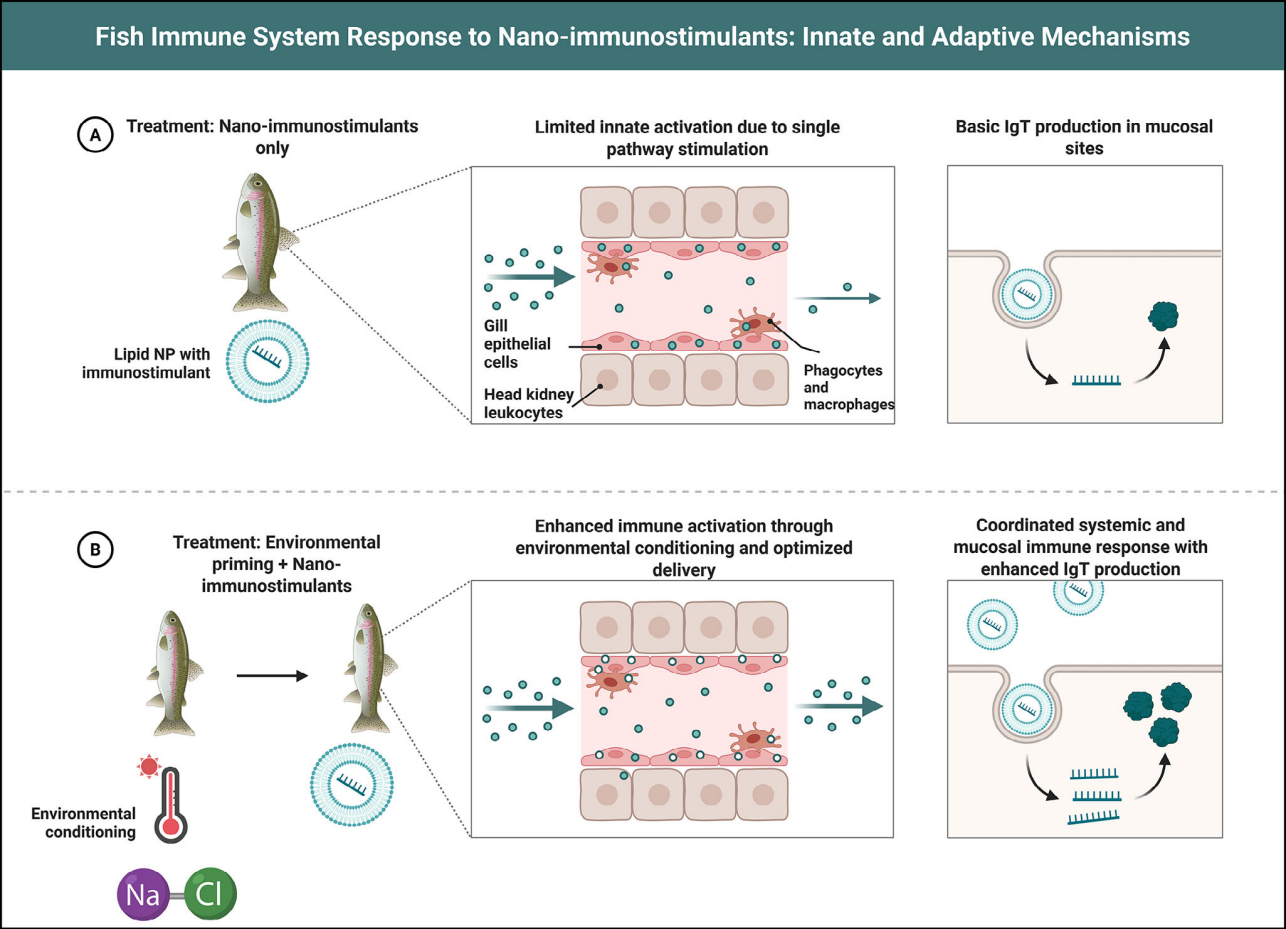
Conceptual illustration contrasting teleost innate and adaptive mucosal‐systemic immune cascades under standard versus environmentally optimised nano‐delivery.

The fish immune system maintains both shared and distinct features with mammalian immune systems, which determines how NISs interact with them. Teleosts depend on their innate defence mechanisms and their organised blood cell production system, which operates in the anterior kidney (head kidney) and spleen, while their mucosal defence occurs in the gill skin and gut. As illustrated in Figure 4, this organisation creates a functional distinction between systemic immune compartments, mainly represented by the anterior kidney/head kidney and spleen, and mucosal immune barriers, including the gills, skin and gut. This distinction is important for NIS design because orally or immersion‐delivered particles first interact with mucosal surfaces such as the gut epithelium, gill epithelium and skin mucus, whereas injected or systemically distributed formulations may more directly influence kidney‐ and spleen‐associated leukocyte responses. Therefore, Figure 4 should be interpreted as a route‐dependent framework showing how NISs can differentially activate mucosal IgT/IgZ‐related responses and systemic kidney–spleen immune coordination. The head kidney and spleen of teleosts contain melanomacrophage centres (MMCs), which perform a similar role to mammalian germinal centres. The mucosal immune response of teleosts depends on B cells and immunoglobulins, especially IgT (Iqbal et al. 2024; Salinas 2015; Steinel and Bolnick 2017; Y. Yu et al. 2020). Research shows that *Nigella sativa* and *Cynara scolymus* and *Pimpinella anisum* L. and *Allium sativum* and glutamine protect against toxic exposures by enhancing haematological and antioxidant and histopathological markers (Çelik et al. 2024; Esen et al. 2025; Öz 2025; Öz and İnanan 2025; Öz, Inanan, Karasahin, et al. 2024; Öz, Inanan, Üstüner, et al. 2024; Öz, Üstüner, et al. 2024; Öz, Üstüner, Jumayeva, et al. 2025; Tok et al. 2025). The anterior kidney functions as the main haematopoietic organ that produces leukocytes for both systemic and mucosal immune defence and the kidney–spleen axis together with MMC‐like structures functions to control humoral responses through structures that resemble mammalian germinal centres but with unique teleost arrangements (Hu et al. 2024; Steinel and Bolnick 2017; Zwollo et al. 2005). The activity of leukocytes together with NP uptake and immunomodulation responds to environmental conditions such as temperature and salinity levels, which produce distribution patterns that depend on both the organ location and specific conditions (Dawood et al. 2020; Smith et al. 2019; Tokunaga et al. 2017). Thus, NP‐immune interactions in fish are shaped by a conserved innate framework along with unique haematopoietic and MMC‐mediated adaptive features, while environmental factors dictate the efficiency of these processes (Iqbal et al. 2024; Salinas 2015; Steinel and Bolnick 2017). The differential immune system responses to NISs under basic treatment versus environmental conditioning combined treatment are depicted in Figure 4.

### 3.2 Nano‐Immune System Interactions

The detection of fish by NPs occurs through pattern recognition receptors (PRRs), which use common pathways yet have special features that exist only in teleost species. The detection of NPs occurs through PRRs, which consist of Toll‐like receptors (TLRs), NOD‐like receptors (NLRs), RIG‐I‐like receptors (RLRs) and C‐type lectins. The detection process activates both MyD88‐dependent and MAVS‐dependent signalling pathways, which result in NF‐κB activation and subsequent interferon and stimulated gene expression (S. N. Chen et al. 2017; Chu and Xu 2020; Mogensen 2009; Takeuchi and Akira 2010). Critically, while these signalling cascades share homology with mammalian models, recent transcriptomic analyses in teleosts have experimentally validated that NPs specifically modulate fish‐unique pathway components—such as distinct TLR isoforms and the IgT/IgZ mucosal immunoglobulin system—confirming that these responses are evolutionarily adapted teleost mechanisms rather than mere projections of mammalian immunology. The cellular uptake process depends on scavenger and mannose receptors and opsonin effects and protein corona formation, which determines how macrophages and monocytes internalise particles through endocytosis (Chao et al. 2013; Patel et al. 2010; F. Zheng et al. 2015). The entry of PRR cells into NPs depends on their surface properties, which include charge, hydrophobicity and protein corona composition, to produce modified cytokines and affect mononuclear phagocyte system (MPS) clearance (Mortimer et al. 2014; Srijampa et al. 2020). The response of teleost species to NPs depends on their PRR diversity and their haematopoietic tissue distribution and MALT‐based mucosal immunity elements, which include IgT/IgZ and mucosal‐associated cells (MCs) (Salinas 2015; Steinel and Bolnick 2017; Y. Yu et al. 2020; Zwollo et al. 2005). The environmental conditions of temperature and salinity and stress levels determine how PRR signalling works and how NPs spread throughout the body, which results in different responses between species (C. Sousa et al. 2022).

### 3.3 Innate Immunity Modulation

NISs produce enhanced and accelerated innate responses in fish compared to conventional stimulants because they enhance the uptake process and receptor binding and signalling in conserved PRR pathways and leverage nano‐specific effects through protein corona formation and targeted delivery (S. P. B. Sousa et al. 2020; Srijampa et al. 2020). The compounds activate macrophages and monocytes better than conventional immunostimulants by using PRRs (TLRs, NLRs and RLRs) and C‐type lectins to increase NF‐κB‐driven cytokine production and by improving endocytosis and cross‐presentation‐like processes, which results in better phagocytosis and respiratory burst and antimicrobial peptide release (Srijampa et al. 2020; Takeuchi and Akira 2010; F. Zheng et al. 2015). The NF‐κB, MAPK and interferon regulatory factor (IRF) family axes function as signalling cascades that TAK1 regulates through its role as an essential upstream activator that controls both cytokine and interferon responses (Wei et al. 2021; Yan et al. 2022). The duration of innate activation depends on NP features and the biological environment because protein corona development leads to short‐term or extended signalling patterns that can spread through exosome‐ or microvesicle‐mediated communication to trigger systemic reactions. The duration of innate activation varies between species and formulation types and lasts between hours and days in teleost models (Y. Liu et al. 2018; Lü et al. 2015; Srijampa et al. 2020). Environmental temperature and salinity affect PRR signalling and biodistribution through their influence on these biological processes (H. Fang et al. 2022; C. Sousa et al. 2022).

### 3.4 Adaptive Immunity Enhancement

NISs may enhance adaptive‐like immune memory in fish by improving antigen uptake, processing and presentation by dendritic‐like cells, macrophages and B cells, thereby supporting B‐cell activation, antibody responses and memory‐associated humoral responses within teleost lymphoid tissues and MMC‐associated immune structures (Angulo et al. 2021; Öz, Üstüner, and Dikel 2026; Popa and Springer 2021; Steinel and Bolnick 2017; Thompson et al. 2023). The combination of NP‐enabled cross‐presentation and dendritic cell maturation leads to strong CD4^+^ T‐helper and CD8^+^ T‐cell responses, which enable long‐term antibody production and memory cell development according to nanovaccine concepts and erythrocyte‐mediated antigen delivery that enhances central memory T cells (Thompson et al. 2023; Ukidve et al. 2020). The use of surface‐coated or cargo‐loaded NPs in antigen presentation pathways through MHC class I/II results in better APC uptake and endosomal escape and costimulation, which produces long‐lasting adaptive immune responses (Nguyen and Tolia 2021; Radhakrishnan et al. 2023). The adaptive outcomes result from species and age factors and vaccination methods and environmental conditions that influence APC distribution, lymphoid organ activity and mucosal immunity, while memory quality depends on IgT/IgZ and B‐/T‐cell repertoires that differ between species and age groups (Angulo et al. 2021; Harshitha et al. 2023; Thompson et al. 2023). Environmental stressors affect memory stability through their impact on PRR signalling and changes in cytokine levels (Angulo et al. 2021; Harshitha et al. 2023; Thompson et al. 2023). However, adaptive immunity in teleosts should not be interpreted as identical to mammalian germinal centre‐dependent memory. Instead, NIS‐induced adaptive enhancement in fish is better understood as a coordinated response involving APCs, B‐cell maturation in kidney and spleen compartments, systemic IgM production and mucosal IgT/IgZ responses, with the magnitude and duration of protection depending on species, age, administration route, antigen type and environmental conditions (Öz and Üstüner 2026b; Öz, Üstüner, Çifci, et al. 2025; Salinas 2015; Steinel and Bolnick 2017; Uribe et al. 2011; Y. Yu et al. 2020; Zwollo et al. 2005).

### 3.5 Immunological Biomarkers and Assessment Methods

Standardised tests that evaluate both innate and adaptive indicators provide the most dependable biomarkers for determining fish reactions to NISs. The essential biomarkers for teleost research consist of total and differential leukocyte counts and measurements of respiratory burst and myeloperoxidase activity and complement activation markers (C3 and alternative pathway) and cytokine transcripts (TNF‐α, IL‐1β and IL‐6), which are present in blood and gill tissues and show rapid responses to nano‐stimulants (Rashidah et al. 2023). The use of nanovaccine‐like formulations enables researchers to monitor adaptive responses through the measurement of serum total Ig and mucosal IgT/IgZ levels (Rashidah et al. 2023). Oxidative stress panels (SOD, CAT, GPx and MDA) are often reported together with antioxidant responses as additional markers of nano‐induced inflammation (Dawood et al. 2018; Moustafa et al. 2021). The use of validated qPCR panels for PRR and NF‐κB‐driven genes together with calibrated ELISAs for immunoglobulins and complement is recommended for standardisation purposes. Species and age differences require researchers to establish reference ranges for each species and age group while conducting experiments under controlled rearing conditions and to collect samples at peak innate response times (6–72 h) followed by humoral response measurements (2–4 weeks) (Dawood et al. 2018). The evaluation of blood, mucus and gill tissue and spleen/head kidney samples requires standardised storage and handling methods to support research comparisons across different studies (Dawood et al. 2018; Rashidah et al. 2023).

## 4 Applications and Case Studies in Commercial Fish Species

### 4.1 Freshwater Species Applications

Nano‐formulations experience major stability issues when they encounter different types of freshwater environments, but their targeted delivery capabilities and dose sparing and mucosal immunity enhancement functions create fresh opportunities for freshwater applications (Ganguly et al. 2010; Ruyra et al. 2013; W. Wang et al. 2016). The stability and effectiveness of freshwater environments depend on three main water chemistry factors: pH levels, water hardness and ionic strength, which affect NP aggregation, protein corona formation and teleost innate cell uptake. This process affects both the safety and effectiveness of the substance (Rodrigues et al. 2020; Ruyra et al. 2013). Species‐ and system‐specific considerations: The immune responses of major freshwater species, including carp, tilapia and rainbow trout, differ from each other because they have unique β‐glucan and chitosan receptor profiles and mucosal IgT/IgZ presence, which requires species‐specific dosing and formulation and administration methods (diet, immersion) for effective use (S. M. Lin et al. 2011; Meena et al. 2013).

### 4.2 Marine Species Applications

**FIGURE 5:**
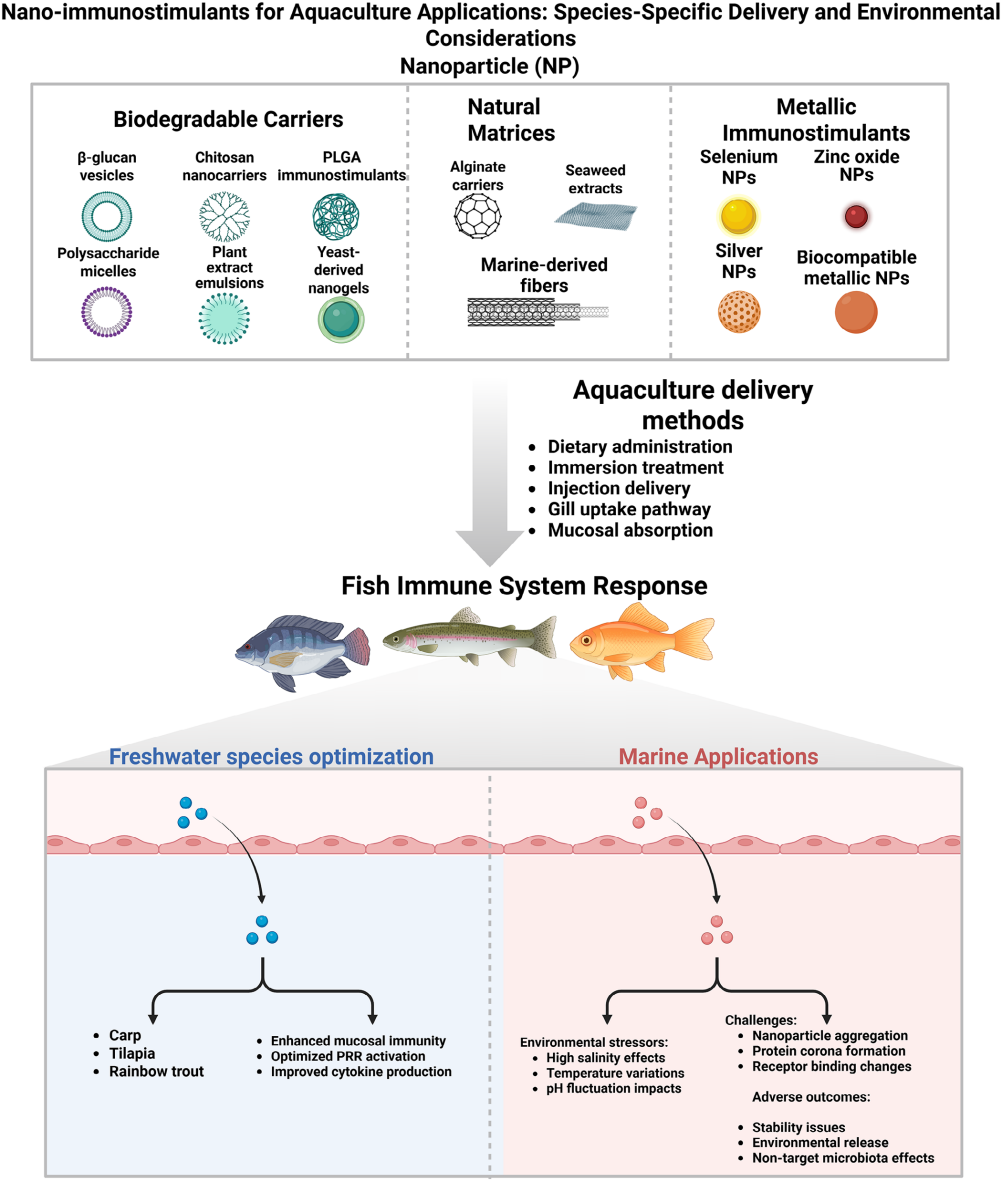
Evidence‐based summary mapping nano‐immunostimulant delivery routes, platform stabilities and efficacy drivers across freshwater and marine systems.

NISs need particular environmental conditions of marine ecosystems to function properly. The stability of NPs depends on salinity, pH and temperature because these factors affect their aggregation, protein corona formation and mucosal transport, which determines their uptake by teleost innate cells and their subsequent signalling pathways (Rodrigues et al. 2020; Ruyra et al. 2013; W. Wang et al. 2016). The stability of NPs in seawater becomes more stable when salt concentrations and ionic strength are high, but these conditions change the corona structure and receptor binding, which affects PRR activation and cytokine production (Rodrigues et al. 2020; Ruyra et al. 2013). For marine applications, coating strategies such as PEGylation, chitosan–alginate multilayers, silica shells, phospholipid coatings and zwitterionic or PVP‐based surface modifications are recommended because they can reduce salt‐induced aggregation, surface corrosion, uncontrolled ion release and protein corona instability in high‐salinity seawater conditions. The changes in temperature influence metabolic rates and leukocyte activity and NP biodistribution patterns, which result in different treatment outcomes between species and body locations (S. M. Lin et al. 2011; Rodrigues et al. 2020). The changes in pH values affect both the surface charge and dissolution rates of materials, which could modify their adjuvant properties and safety characteristics when used in gill and gut mucosa applications (Rodrigues et al. 2020; Ruyra et al. 2013). The use of marine‐based matrices in drug delivery systems faces three major challenges, which include fouling and environmental release issues, and non‐target microbiota effects and regulatory compliance problems that can be addressed through environmentally friendly matrices and controlled‐release systems and thorough ecotoxicity assessments (Cirivello et al. 2025; Ganguly et al. 2010; Ruyra et al. 2013; W. Wang et al. 2016). The unique characteristics of each species emerge from their distinct mucosal immune systems, which include IgT/IgZ antibodies and their different haematopoietic organ locations and NP receptor diversity (S. M. Lin et al. 2011; Meena et al. 2013; Rodrigues et al. 2020). Figure 5 provides a comprehensive overview of NIS applications in aquaculture systems, highlighting species‐specific delivery methods and environmental considerations for both freshwater and marine applications. A comprehensive summary of experimental trials involving various fish species, dosages and target pathogens, illustrating the immunomodulatory efficacy of nano‐formulations, is provided in Table 2.

**TABLE 2:** Summary of experimental trials utilising nanotechnology‐based immunostimulants, organised according to administration route where applicable, including dietary/feed, immersion/bath, oral and injection applications, and their impacts on growth performance and immune responses in fish and crustacean species.

Nanoparticle type/formulation	Fish/crustacean species	Dose and duration	Target pathogen/challenge	Key immunological and protection outcomes	References
Chitosan–selenium nanoparticles (CTS‑SeNP) in diet	Zebrafish (*Danio rerio*)	5–20 µg Se/g diet; best at 10 µg/g; 3–60 days	*Aeromonas hydrophila* (ip injection)	↑ lysozyme, respiratory burst, splenocyte proliferation; rapid (3–9 days) and sustained (to 60 days) immunostimulation; ∼26.7% higher survival than control	Xia et al. (2019)
ZnONPs + SeNPs (dietary)	Rohu (*Labeo rohita*)	ZnONP 10 mg/kg + SeNP 0.3 mg/kg diet; 120 days	*A. hydrophila*	↑ respiratory burst, lysozyme, myeloperoxidase; altered serum enzymes; relative percent survival (RPS) ∼60% vs. 45% control	Swain et al. (2019)
ZnO nanoparticles in feed	Nile tilapia (*Oreochromis niloticus*)	30 or 60 µg/g diet for 8 weeks	*A. hydrophila* (immersion/injection)	↑ phagocytosis, serum antibacterial, lysozyme, respiratory burst; upregulation of IgM, TNF‐α, IL‐1β, antioxidant genes; higher survival and disease resistance at 30–60 µg/g	Sherif et al. (2023)
ZnONPs vs. organic ZnO (dietary)	African catfish (*Clarias gariepinus*)	20 or 30 mg/kg diet (ZnO or ZnONPs); 60 days	*Pseudomonas aeruginosa* (ip 3 × 10^7^ CFU/mL)	20 mg/kg ZnONPs: best growth, FCR, ↑ IgM, lysozyme, NO, antioxidant status; improved survival after challenge	Mahboub et al. (2020)
Exopolysaccharide‐mediated ZnONPs (EPS‐ZnONPs) in diet	Mozambique tilapia (*Oreochromis mossambicus*)	2, 5, 10 mg/g diet; 30 days	*A. hydrophila* and *Vibrio parahaemolyticus* (ip 50 µL; 1 × 10^7^ cells/mL)	At 10 mg/g: best growth, ↑ serum/mucus cellular and humoral immunity, ↑ SOD, CAT, GPx; reduced mortality to both bacteria	Abinaya et al. (2023)
β‐1,3‐Glucan‐binding protein–ZnONPs (Ppβ–GBP–ZnONPs) in diet	Mozambique tilapia (*O. mossambicus*)	0.001%–0.004% in diet; 30 days	*A. hydrophila* (1 × 10^7^ CFU/mL ip)	0.004%: highest myeloperoxidase, lysozyme, ROS, complement, antiprotease, ALP; strong antibiofilm; markedly reduced mortality	Anjugam et al. (2018)
Plant‐mediated ZnONPs and SeNPs (Mb–ZnONPs, Mb–SeNPs)	Mozambique tilapia (*O. mossambicus*)	0.5, 1, 2 mg/kg diet; duration not explicitly stated (growth + challenge phase)	*A. hydrophila*	Mb–SeNPs (2 mg/kg): highest SOD, CAT, GPx, reduced lipid peroxidation; ↑ MPO, RBA, lysozyme, complement; fish less prone to infection	Yazhiniprabha et al. (2022)
Commercial vs. green‐synthesised ZnONPs (waterborne)	Common carp (*Cyprinus carpio*)	25% or 50% of 96 h LC_50_; 21 days	No experimental pathogen; immunotoxicity profile	Green ZnONPs caused less immunosuppression: higher lysozyme and complement vs. commercial NPs; indicated safer immunological profile	Rashidian et al. (2021)
Seaweed‐synthesised ZnONPs (*Sargassum wightii*) via immersion and diet	Green tiger shrimp (*Penaeus semisulcatus*)	Immersion and dietary exposure; multiple doses over experimental period	Not specific bacterial challenge in shrimp; antibiofilm vs. *Bacillus subtilis*, *Staphylococcus aureus*, *Shigella sonnei*, *P. aeruginosa*	ZnONPs ↑ total haemocyte count, respiratory burst, phenoloxidase, SOD; strong antibiofilm; proposed as bacteriostatic immunostimulant	Ishwarya et al. (2018)
ZnO–*Ulva lactuca* nanocomposite (ZnO‑Ul NC) in diet	Red swamp crayfish (*Procambarus clarkii*)	Low and high doses (ZnO‐Ul L, ZnO‐Ul H) for 90 days	No explicit challenge; antibacterial tests vs. *Citrobacter*, *Enterobacter*	ZnO‐Ul NC: highest haemocyte counts, phenoloxidase, NO, SOD, GSH and upregulated proPO, SOD, lysozyme; strong immunostimulation	Omar et al. (2024)
Gold nanoparticles (AuNP; ∼18.6 nm) in feed	Shrimp (*Litopenaeus vannamei*)	Single oral dose 0.2, 2, 20 µg/g feed; 6 days	*V. parahaemolyticus* (AHPND)	2 µg/g: ↑ immune gene expression early (24–48 h), no toxicity; ∼80% survival and reduced tissue lesions vs. control	Tello‐Olea et al. (2019)
ZnONPs—shrimp bath and dietary	*P. semisulcatus*	Immersion and feed, doses up to 100 µg/mL (also larval mosquito tests)	Broad aquatic bacteria; no specific fish pathogen challenge	Marked increases in shrimp THC, RB, PO, SOD; strong antibiofilm and insecticidal activity; proposed as immunostimulant	Ishwarya et al. (2018)
Curcumin‑loaded chitosan NPs (Cur‑CSNPs) in diet	White leg shrimp (*L. vannamei*)	Cur‐CSNPs in feed (multiple levels) during growth and challenge periods	*Vibrio harveyi*	In challenged and non‑challenged shrimp: ↑ SGR, DGC, survival, haemocyte count, PO, SOD; ↑ lysozyme, cMnSOD, lectin mRNA; improved resistance	Bhoopathy et al. (2021)
Bee venom‑loaded chitosan NPs (BV‑CSNPs) in diet	White leg shrimp (*L. vannamei*)	0.1, 0.2, 0.3 mg BV‐CSNPs/kg feed; 63 days	*V. parahaemolyticus*	Dose‑dependent ↑ growth, feed efficiency, haemocytes, PO, lysozyme, phagocytosis, antioxidant and digestive enzymes; lower mortality post‑challenge	Eissa et al. (2025)
Dietary chitosan nanoparticles (CSNPs)	Rainbow trout (*Oncorhynchus mykiss*)	5 g/kg feed; 21 days prophylactic or 21 + 28 days therapeutic	*Yersinia ruckeri* (ERM disease)	CSNPs improved serum/mucus bactericidal activity, intestinal histology and expression of IL‐1β, TGF‐β, LYZ II, IgM; therapeutic regimen gave higher resistance and lower morbidity	F. Ahmed et al. (2021)
Dietary chitosan nanoparticles (CSNPs), preventive vs. therapeutic	Rainbow trout (*O. mykiss*)	CSNP diet for 21 days (preventive) or 21 days pre‐ + 28 days post‐challenge (therapeutic)	Enteric redmouth (*Y. ruckeri*)	Therapeutic CSNPs: RPS 80% vs. positive control; sustained upregulation of inflammatory genes, preserved splenic reticulin and innate immunity	Saleh et al. (2022)
Chitosan nanoparticles (CNP) in diet	Rohu (*L. rohita*)	0, 0.25, 0.5, 1, 2 g/kg diet; 20 days	*A. hydrophila*	1 g/kg: highest respiratory burst, lysozyme, MPO, catalase; in vitro antibacterial vs. *A. hydrophila*; highest RPS in challenge test	Naveen Kumar et al. (2022)
Chitosan nanoparticles (CNPs; ∼181 nm, +37 mV) in water	Zebrafish larvae	5 µg/mL exposure (5 dpf onward)	*A. hydrophila* (bath infection)	5 µg/mL CNPs: upregulation of many immune genes (IL‐1β, TNF‐α, IL‐6, IL‐10, chemokines, lysozyme, defensin, IFN‐related) and ↑ survival vs. control; higher doses more toxic	Nikapitiya et al. (2018)
Ulvan‐loaded chitosan NPs (CS‐UL‐TPPNPs) in diet	Senegalese sole (*Solea senegalensis*)	Oral for 30 days	*Photobacterium damselae* subsp. *piscicida* (in vitro); no in vivo challenge	CS‐UL‐TPPNPs: higher in vitro inhibition of *P. damselae*; in vivo ↑ serum lysozyme and expression of il1b, il6, hamp1, tf, c3 vs. controls	Ponce et al. (2024)
Nanoliposomes loaded with LPS + poly I:C (NL‑LPS/poly I:C)	Zebrafish larvae	Bath immersion at 4 dpf; single or repeated exposures	*A. hydrophila* (bath immersion model)	Nanoliposomes ingested and accumulated in intestine; strong upregulation of innate genes (tnfα, il1β, nos2a, irf1a, ptgs2a); protected larvae from lethal *A. hydrophila*, whereas free immunostimulants or empty NPs did not	Ji et al. (2019)
Phytoglycogen NPs complexed with dsRNA (poly I:C; HMW‐NP) in feed	Rainbow trout (*O. mykiss*)	Oral gavage or coated pellets with HMW‐NP vs. free poly I:C	No specific pathogen; antiviral innate response model	HMW‐NP induced higher IFN1 and ISGs (vig‐3, Mx1) in intestine and head kidney than free poly I:C, indicating stronger local and systemic antiviral priming	Samms et al. (2022)
Carboxymethyl chitosan nano‑peptide CMCS‑20H in diet	Fish (species in study: Chinese grass carp model species, see abstract)	Oral for 42 days	Bacterial pathogen (noted as common fish pathogen; challenge after feeding)	CMCS‐20H NPs: ↑ complement C3, lysozyme, SOD; highest survival (46% vs. 28% control), lower tissue bacterial loads; ↑ goblet cells and mucin, upregulation of multiple cytokines (IL‐1β, IL‐6, TNF‐α, IL‐2, IFN‐γ2, IgM)	Huo et al. (2022)
CMCS‐20a nano‐cytokine (CXCL20a) in feed	Grass carp (*Ctenopharyngodon idella*)	28 days oral (low‐temperature vacuum‐ vs. spray‐dried formulations)	Grass carp reovirus (GCRV)	Low‐temperature CMCS‐20a: highest survival, ↑ complement C3, lysozyme, SOD; preserved goblet cells/mucin; ↑ IFN1, Mx2, Gig1, IgM; reduced viral load and tissue lesions	Z. Wang et al. (2022)
Chitosan NPs as mucosal vaccine carriers	Multiple teleost species	Various (typically µg–mg chitosan per g fish, oral or bath)	Bacterial and viral pathogens (multiple)	Across trials: enhanced mucosal immunity (lysozyme, IgM, gene expression) and improved survival; systematic review notes wide dose range and inconsistent reporting of chitosan properties	F. Ahmed et al. (2019); Collado‐Gonzalez and Esteban (2022); Swain et al. (2019)
Nano‐curcumin vs. free curcumin in diet	Nile tilapia (*O. niloticus*)	50–200 mg/kg nano‑curcumin vs. free; 65 days	Heat stress (40°C); no pathogen	Nano‐curcumin (100 mg/kg) best for growth, immune markers (IgM, C3, C4) and lower cortisol/glucose under stress; supports role as anti‐stress immunonutrient	Abdel‐Ghany et al. (2023)
Curcumin nanoparticles (C‐NPs) in diet	Nile tilapia (*O. niloticus*)	15–60 mg/kg diet; 60 days	No pathogen; physiological evaluation	Dose‐dependent ↑ growth, digestive enzymes, RBC/WBC, antioxidant enzymes, lysozyme, total Ig; improved liver and intestinal histology	Abdel‐Tawwab et al. (2022)
*Astragalus membranaceus* nanoparticles (ANP) in diet	Nile tilapia (*O. niloticus*)	1% or 2% ANP/kg diet; 4 weeks	*Aeromonas veronii*; cold and hypoxia stress	ANP significantly ↑ lysozyme, NO, SOD, CAT, GPx; ↓ ALT, AST, glucose, cortisol; improved growth and markedly lower mortality after *A. veronii* and stress challenges	Elabd et al. (2020)
Chitosan–vitamin C/E nanoparticles (CCE‐NPs) in diet	Nile tilapia (*O. niloticus*)	CCE‑NPs fed 7 or 14 days pre‑ and/or post‑salt bath (30 ppt, 30 min/day)	*Streptococcus agalactiae* after salt‐stress	CCE‐NPs blunted cortisol/glucose rise, restored mucus lysozyme and antibacterial activity faster, ↑ GPx, SOD, CAT expression; reduced mortality vs. control during *S. agalactiae* infection	Elnagar et al. (2024)
Quercetin‐loaded mesoporous silica NPs (QMSNs) in diet	Nile tilapia (*O. niloticus*)	0.25, 0.50, 1 g/kg diet; 90 days	*Klebsiella pneumoniae*, *Streptococcus*	0.50 g/kg: best digestive enzymes, phagocytic index, respiratory burst, serum antibacterial, ↑ SOD and CAT genes; significantly highest survival after 15‐day co‐challenge	Shameem et al. (2023)
Platinum NPs + shrimp lectin hybrid (Pt‐lec) (injection)	Nile tilapia (*O. niloticus*)	Therapeutic injection after *A. hydrophila* infection	*A. hydrophila*	Pt‑lec rapidly reduced bacterial load (< 12 h), normalised fish condition; ↑ Myd88, COX‑2 expression (immune activation); proposed as rescue immunostimulant vs. antibiotics	Lakshmi et al. (2023)
Chlorella vulgaris extract‐coated magnetic iron oxide NPs (ChVE@Mag‐iron NPs) in diet	Nile tilapia (*O. niloticus*)	90 days; ChVE, Mag‐iron NPs, or ChVE@Mag‐iron NPs	*Ichthyophthirius multifiliis*, then *A. hydrophila* co‐infection	ChVE@Mag‐iron NPs: best growth, ↑ skin/gill antioxidant enzymes, serum immunity (lysozyme, IgM, ACH, MPO), ↓ white spot load by 92%, ↓ mortality by 84% vs. control; lowest *A. hydrophila* loads	D. Ibrahim et al. (2024)
Protein ‘nanopellets’/inclusion‐body NPs (viral antigens)—oral or injection	Zebrafish, rainbow trout; VHSV models	Various in vitro and in vivo oral/injection protocols	Viral haemorrhagic septicaemia virus (VHSV) and other viruses	Viral antigen NPs taken up by cells and intestine; upregulate antiviral genes (IFNs, ISGs) and induce specific anti‐VHSV IgM; 2025 in vivo trial shows neutralising antibodies and boosted titres after infection	Thwaite et al. (2025); Thwaite et al. (2018); Torrealba et al. (2024)

*Note*: Administration routes are indicated within the formulation and dose/duration columns and include dietary/feed, oral, immersion/bath exposure and injection‐based applications.

The studies summarised in Table 2 indicate that NISs can enhance innate immune markers such as lysozyme activity, respiratory burst, phagocytosis, complement activity, antioxidant enzymes and cytokine expression. However, the strength of evidence varies. Chitosan‐based, selenium‐based and selected zinc oxide formulations have been tested in multiple fish species and pathogen models, whereas lipid‐based, silica‐based, carbon‐based and hybrid systems remain less extensively validated. In addition, many studies rely on short‐term laboratory challenge models and relatively few evaluate long‐term growth performance, residue safety, environmental release, microbiome effects or farm‐scale survival. Therefore, current evidence supports promising experimental efficacy but remains insufficient for broad commercial recommendations without field validation.

### 4.3 Performance Parameters Evaluation

The field depends on established biomarkers and validated protocols to enable comparison of NIS effects between different research studies. Standardised tests that combine natural and learned responses through leukocyte profiles and respiratory burst and lysozyme and myeloperoxidase and complement activity (ACH_50_/C3) and transcriptional panels for PRRs/NF‐κB pathways and cytokines (TNF‐α, IL‐1β and IL‐6) in blood and mucosal tissues and humoral markers (total immunoglobulin and mucosal IgT/IgZ) and oxidative stress panels (SOD, CAT, GPx and MDA) provide the basis for reliable evaluation (Dawood et al. 2018; Rashidah et al. 2023). The standardisation process needs validated qPCR panels for fundamental genes and ELISAs for immunoglobulins and complement standardised sample management for blood and mucus and gill and spleen/head kidney samples (Dawood et al. 2018; Rashidah et al. 2023). The sampling schedule requires knowledge of innate response peak patterns that appear between 6 and 72 h followed by humoral response development between 2 and 4 weeks and memory response evaluation after booster doses when needed (Dawood et al. 2018). The translation of economic data requires researchers to link biomarker‐based performance enhancements to commercial metrics that evaluate growth and feed efficiency and disease resistance, and studies show that immunostimulants enhance fish immune system function (Q. Wang et al. 2024). The evaluation of long‐term health effects requires long‐term research studies that monitor how immunomodulation and tolerance formation and microbiota‐body connections evolve after the initial treatment period (Abarike et al. 2019; Akhter et al. 2015).

### 4.4 Disease Resistance Enhancement

**FIGURE 6:**
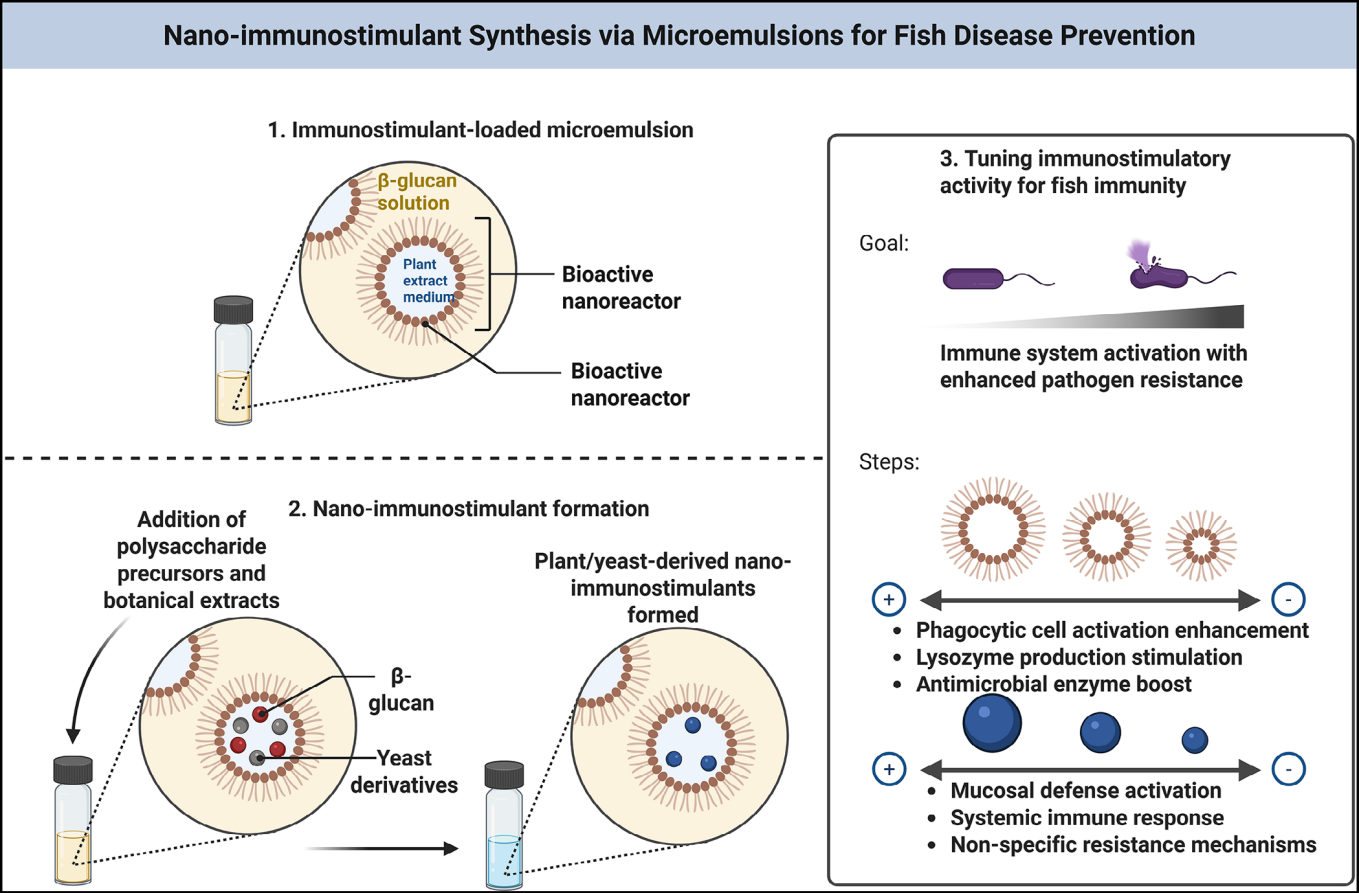
Conceptual illustration of the microemulsion synthesis route and its subsequent activation pathways for targeted fish disease prevention.

NISs boost fish resistance through their power to strengthen both innate and non‐specific immune defences, which fight against different aquaculture pathogens. The system operates through three mechanisms that activate phagocytic cells and boost lysozyme and antimicrobial enzyme production and immune gene expression to enhance the body's ability to detect and eliminate pathogens at the beginning of an infection (Abarike et al. 2019; Ching et al. 2020; Z. Zhang et al. 2019). Plant‐ and yeast‐derived immunostimulants (e.g., β‐glucans, polysaccharides and botanicals) activate both mucosal and systemic defences by using non‐specific resistance mechanisms that support pathogen‐targeted vaccines (Barros et al. 2015; Khanjani et al. 2022; Machuca et al. 2022). Medicinal and aromatic plant oils (MAPOs) have also been shown to support growth, modulate immune responses and improve stress tolerance, although effects may vary with oil composition and dosing (Öz, Üstüner, and Çifci 2026; Üstüner et al. 2026). NISs provide fast and broad protection against pathogens, but their protection duration remains shorter than that of vaccines, and they do not target specific pathogens (Barros et al. 2015; Petit and Wiegertjes 2016; Z. Zhang et al. 2019). The immune system responds to β‐glucans through long‐term innate reactions, but vaccines generate specific protection through complex development and administration processes that result in extended protection (Álvarez‐Rodríguez et al. 2018; Petit and Wiegertjes 2016). Recently, the integration of phytotherapy, polyphenols (e.g., quercetin), natural extracts (e.g., ginger, apple cider vinegar, cornelian cherry) and probiotics (*Lactobacillus fermentum*, *Lactobacillus rhamnosus* and *Saccharomyces boulardii*) into aquaculture has gained significant attention for their ability to enhance non‐specific immunity, upregulate antioxidant gene expression and improve resistance against major pathogens such as *Aeromonas hydrophila* and *Lactococcus garvieae*. Moreover, combining these eco‐friendly natural additives with advanced nano‐formulations represents a highly promising and sustainable intervention tool to maximise their bioavailability, improve water quality and further strengthen the host defence system without the adverse effects of conventional antibiotics (Ahmadifar, Dawood, et al. 2019; Ahmadifar, Moghadam, et al. 2019; Ahmadifar et al. 2022; Ahmadifar, Sheikhzadeh, et al., 2019; Ahmadifar et al. 2026; Ahmadifar et al. 2021; Armobin et al. 2023; Fajardo et al. 2022; Hosseinzadeh et al. 2025; Jeyavani et al. 2022; Kolupula et al. 2024). Furthermore, functional feed additives such as artichoke (*C. scolymus*) leaf extract have demonstrated significant efficacy as metabolic stabilisers, effectively mitigating chemical‐induced oxidative stress, haemato‐biochemical disruptions and structural tissue degeneration in aquaculture species (Gümüş et al. 2026). NISs work best as additional disease prevention methods that need to be used alongside vaccines and biosecurity measures (Bagni et al. 2005; Barros et al. 2015; Giri et al. 2018). The synthesis of NISs via the microemulsion technique for enhanced fish disease prevention is illustrated in Figure 6. To provide a structured and comprehensive cross‐examination of these diverse empirical findings, a detailed comparative synthesis mapping various NP platforms, their targeted aquaculture species, administration routes, immunological indicators and associated toxicological trade‐offs is systematically summarised in Table 3.

**TABLE 3:** Comprehensive comparative synthesis of nano‐immunostimulant platforms, target aquaculture species, administration routes, immunological outcomes and practical safety trade‐offs.

Nanoparticle type/platform	Target aquaculture species	Administration route and dosage	Immunological outcomes (innate and adaptive biomarkers)	Disease resistance outcomes (target pathogens and survival metrics)	Potential toxicity and bioaccumulation concerns	Practical advantages and limitations	References
ZnO nanoparticles (dietary)	Nile tilapia (*Oreochromis niloticus*)	Feed, 30 or 60 µg/g for 8 weeks	Increased phagocytic activity, serum antibacterial activity, lysozyme, respiratory burst and broad immune‐gene upregulation including *IgM*, *TNF‐α*, *IL‐1β*, *IL‐10*, *HSPs*, *SOD* and *CAT*	Higher survival after *Aeromonas hydrophila* challenge in both 30 and 60 µg/g NP groups	This efficacy study did not report overt toxicity, but separate exposure work shows ZnONPs can accumulate in aquatic systems and depress immunoglobulins, lysozyme, nitric oxide and myeloperoxidase under waterborne exposure	Strong antibacterial and immunostimulatory signal, but therapeutic window depends heavily on route, dose and formulation; feed inclusion appears beneficial whereas environmental exposure can be immunotoxic	Khalil et al. (2023); Sherif et al. (2023)
ZnO nanoparticles vs. zinc acetate	Nile tilapia (*O. niloticus*)	Feed, 20, 40, or 60 mg/kg ZnONPs for 8 weeks; comparator 40 mg/kg zinc acetate	Best overall immune and oxidative profile at 40 mg/kg, including IgM, lysozyme, phagocytosis, respiratory burst, total protein and antioxidant markers	40 mg/kg ZnONPs gave the lowest mortality after *Staphylococcus aureus* challenge	No major toxicity signal stated in this feeding study, but benefit was dose‐dependent and not linearly improved at 60 mg/kg	Suggests nano‐zinc can outperform conventional zinc salts as a feed supplement, but optimisation is species‐ and dose‐specific	Yaqub et al. (2023)
EPS‐mediated ZnO nanoparticles	Mozambique tilapia (*Oreochromis mossambicus*)	Feed, EPS or EPS‐ZnONPs at 2, 5 or 10 mg/g	Improved serum and mucus cellular and humoral immunity plus antioxidant enzymes GPx, SOD and CAT, strongest at 10 mg/g	Lower death rate after *A. hydrophila* and *Vibrio parahaemolyticus* challenge; highest post‐challenge survival in supplemented fish	No explicit bioaccumulation analysis reported	Hybridising probiotic EPS with ZnO appears to enhance efficacy, but evidence is still from controlled laboratory feeding studies only	Abinaya et al. (2023)
Green vs. chemically synthesised ZnO nanoparticles	Common carp (*Cyprinus carpio*)	Waterborne, 25% and 50% of 96 h LC_50_ for 21 days	Green ZnONPs preserved mucus lysozyme and ACH_50_ better than chemically synthesised ZnONPs, indicating lower immunosuppression	No pathogen challenge in this study	Clear safety contrast: green ZnONPs had higher LC_50_ and less immunotoxic effect; all exposed groups showed reduced total protein and some immune depression at higher exposure	Useful caution that synthesis route changes safety profile; this is an exposure‐toxicology study, not a prophylactic efficacy trial	Rashidian et al. (2021)
ZnO‐*Ulva lactuca* nanocomposite	Red swamp crayfish (*Procambarus clarkii*)	Feed, low and high dose for 90 days	Increased total haemocyte count, granular cells, phenoloxidase, NO, SOD, GSH and upregulated proPO, SOD and lysozyme genes, strongest in high‐dose nanocomposite group	Inhibited *Citrobacter freundii* and *Enterobacter hormaechei* in vitro, but no in vivo survival challenge reported	No explicit toxicity or tissue‐residue assessment reported	Cost‐effective and easily synthesised, but disease‐protection evidence remains indirect without a pathogen challenge model	Omar et al. (2024)
Selenium nanoparticles (green/biogenic)	Nile tilapia (*O. niloticus*)	Feed, 0.75 or 1.5 mg/kg for 8 weeks	Increased IgM, lysozyme, reduced glutathione and catalase without inducing pro‐inflammatory cytokine responses; histology remained normal	No pathogen challenge in this specific study	Strong short‐term safety signal: no inflammatory response and no hepatic or splenic histopathological deviation	Promising as a low‐dose micronutrient immunostimulant with a cleaner safety profile than many metal NPs, although direct disease‐resistance evidence is missing here	Al‐Wakeel et al. (2024)
Selenium nanoparticles (dietary challenge study)	Nile tilapia (*O. niloticus*)	Feed, 0.5, 1.0 or 1.5 mg/kg for 60 days	Improved haemato‐biochemical indices, antioxidant capacity and digestive enzymes, with best performance at 1.0 mg/kg	Mortality decreased after *Aspergillus flavus* challenge, especially at 1.0 mg/kg	Histopathology improved rather than worsened at the optimal dose, but long‐term residue studies were not provided	Supports a narrow optimal range approximately 1 mg/kg; over‐ or underdosing may reduce benefit	Eissa et al. (2024)
Silver nanoparticles (dietary)	Rohu (*Labeo rohita*)	Feed, 10, 15 or 20 µg/kg for 56 days	Upregulated IL‐8 and COX‐2, altered NBT, and improved protective immune response, with strongest net benefit at 15 µg/kg	15 µg/kg improved post‐challenge survival to 90% against *A. hydrophila*	Dose ceiling is important: higher dietary levels reduced growth and caused kidney, liver and gill lesions	Potent at low doses but safety margin appears narrow, making formulation control essential	Popoola et al. (2023)
Silver nanowires	White shrimp haemocytes/shrimp aquaculture context	In vitro exposure up to 1000 mg/L	Upregulated LGBP and Toll expression in haemocytes, indicating innate immunostimulation	Antibacterial activity against *V. parahaemolyticus*, *Vibrio alginolyticus* and *Vibrio harveyi* based on MIC assays; no in vivo survival trial	Haemocyte viability stayed above 90% up to 1000 mg/L in vitro	Useful proof‐of‐concept for shrimp disease management, but evidence is preclinical and not yet validated in feeding or farm trials	Sahoo et al. (2026)
Chitosan nanoparticles (therapeutic feed additive	Rainbow trout (*Oncorhynchus mykiss*)	Dietary preventive or therapeutic administration for 21 days before and 28 days after challenge	Increased inflammatory‐mediator gene expression and preserved splenic reticulin integrity, consistent with heightened innate responsiveness	Therapeutic CSNP group achieved 80% relative percent survival in infection challenge	Chitosan nanoparticles are described as low‐cytotoxic and haemocompatible	Strong evidence for polymeric immunotherapy in fish, although the abstract does not specify the exact challenge pathogen in the indexed text	Saleh et al. (2022)
Low‐molecular‐weight chitosan nanoparticles/degraded chitosan	Nile tilapia (*O. niloticus*)	Feed: NCS 0.50%; RCS 0.025%, 0.050% or 0.075%	Enhanced ifng1, nfκb, tnf, lysozyme, IgM, bactericidal activity, reduced MDA and increased GSH/GR; RCS worked at ∼10‐fold lower concentration than NCS	RCS‐0.050 and RCS‐0.075 gave the highest survival and RPS after *Edwardsiella tarda* challenge	No adverse growth effects were observed	Strong evidence that nanoparticle engineering can improve potency and reduce required dose for chitosan‐based feeds	Rattanawongwiboon et al. (2025)
Chitosan neem nanocapsule	Nile tilapia (*O. niloticus*)	Water treatment, 1 mg/L for 7 days	Restored catalase, GSH, lysozyme, nitric oxide and complement C3 in *Aeromonas sobria*‐infected fish	Improved survivability against *A. sobria*; infected untreated fish had 60% survival	No direct residue analysis reported, but CNNC showed aqueous stability and no obvious aggregation issues in characterisation	Useful nonfeed, water‐treatment platform with direct antibacterial activity, but evidence is still short‐term and species‐specific	R. E. Ibrahim et al. (2023)
Ulvan‐loaded chitosan nanoparticles	Senegalese sole (*Solea senegalensis*)	Oral administration	Increased lysozyme and expression of il1b, il6, hamp1, tf and c3, whereas plain chitosan NPs did not significantly differ from controls	In vitro inhibition of *Photobacterium damselae* subsp. *piscicida* stronger than plain chitosan particles at low concentrations; no in vivo survival challenge reported	No toxicity statement reported in indexed text	Adding ulvan appears to materially improve chitosan NP immunostimulation, but challenge efficacy remains untested	Ponce et al. (2024)
Essential oil‐loaded chitosan nanoparticles	Siberian sturgeon (*Acipenser baerii*)	Dietary supplementation	Increased lysozyme, IgM, respiratory burst, ACH_50_ and immune‐gene expression	No pathogen challenge reported	No toxicity concern reported in indexed text	Demonstrates chitosan's utility as a carrier that can potentiate plant‐derived immunostimulants, but protection data are lacking	Adel et al. (2024)
Bee venom‐loaded chitosan nanoparticles	Pacific white shrimp (*Litopenaeus vannamei*)	Feed, 0.1, 0.2 or 0.3 mg/kg for 63 days	Increased total haemocytes, phenoloxidase, lysozyme, phagocytic activity, SOD, CAT and proPO‐related genes LGBP, PX and ppA	Lower mortality after *V. parahaemolyticus* challenge	Histology remained normal with improved hepatopancreatic and intestinal structure	Strong shrimp feed‐additive evidence for chitosan as a delivery scaffold for bioactives	Eissa et al. (2025)
Chitosan polymer‐based immersion nanovaccine	Red tilapia (*Oreochromis* sp.)	Immersion vaccination	Increased specific IgM and bactericidal activity in both serum and mucus, plus higher lysozyme, ACH_50_ and total Ig	RPS 71% after heterologous *Aeromonas veronii* challenge, vs. 15.12% for formalin‐killed vaccine	Safe during vaccination with no mortalities reported	One of the stronger mucosal‐vaccine demonstrations; supports chitosan as a practical immersion‐delivery platform	Sukkarun et al. (2024)
CMCS nanopeptide	Fish species not specified in indexed abstract text	Oral administration for 42 days	Increased serum C3, lysozyme, total SOD, intestinal mucin and goblet cells, and upregulated IL‐1β, IL‐6, TNF‐α, IL‐2, IFN‐γ2 and IgM	Highest survival after *A. hydrophila* challenge at 46% vs. 28% in controls, with lower tissue bacterial load	Negligible toxicity reported	Shows polymer‐peptide nanoparticles can combine antimicrobial and immunopotentiating functions, but protection remained partial rather than near‐complete	Huo et al. (2022)
dsRNA‐phytoglycogen nanoparticle complex	Rainbow trout (*O. mykiss*)	Oral gavage or coated pellets	Higher IFN1 and interferon‐stimulated genes vig‐3 and Mx1 in intestine and head kidney than dsRNA alone, indicating improved local and systemic innate antiviral activation	No direct pathogen survival challenge reported	No toxicity outcome reported	Strong delivery proof‐of‐concept for oral antiviral immunostimulation, but protective efficacy remains inferential without challenge data	Samms et al. (2022)
Dual‐targeted chitosan‐PLGA polymer nanovaccine	Common carp/cyprinids	Vaccine delivery	Efficient APC maturation, high immune‐gene expression, antigen‐specific IgM, and SVCV‐neutralising antibody titres	∼84% survival after SVCV challenge	Good in vivo and in vitro biocompatibility reported	Strong adaptive‐immunity evidence for smart polymeric nanovaccines, although complexity may raise manufacturing costs	C. Zhang, Zhang, et al. (2022)
Calcium phosphate nanoparticle oral vaccine	Nile tilapia (*O. niloticus*)	Oral vaccination	Improved specific IgM, opsonophagocytic killing and immune‐gene expression vs. soluble OMP vaccine	RPS 85.7% against virulent *A. hydrophila*	No toxicity issue reported in indexed text	Non‐invasive vaccination platform with strong efficacy in tilapia, but dosage and longer‐term field practicality remain unclear from the indexed text	Abhiram et al. (2025)

## 5 Comparative Advantages, Limitations and Translational Relevance of NISs

NISs offer several theoretical and experimental advantages over conventional immunostimulants, including payload protection, controlled release, improved mucosal uptake, dose sparing and targeted immune activation. However, these benefits are not universal and depend on formulation type, particle size, surface chemistry, dose, route of administration, host species, water chemistry and pathogen challenge model. Therefore, the practical value of NISs should be assessed through a risk–benefit framework that considers efficacy, toxicity, environmental fate, manufacturability, regulatory feasibility and cost‐effectiveness.

### 5.1 Controlled and Sustained Release Mechanisms

The protective shell of nano‐encapsulation systems maintains immunostimulants throughout stomach passage while delivering them specifically to the digestive tract through pH‐sensitive or enzyme‐degradable or biodegradable materials, which improves the stability and availability of bioactive compounds in fish feed (Dezfooli et al. 2019; Giri et al. 2021). In oral delivery, acid‐resistant NP coatings can protect encapsulated immunostimulants from acidic gastric conditions, enzymatic degradation and premature leaching in the stomach, allowing a larger fraction of the active compound to reach the posterior intestine or hindgut. At this site, changes in pH, digestive enzymes, mucus interactions and microbiota‐associated degradation can trigger gradual NP disassembly and controlled release, thereby enhancing uptake by intestinal epithelial cells, mucus‐associated immune cells and gut‐associated lymphoid tissues that are important portals for pathogen entry in fish. The release patterns of encapsulated substances depend on the production methods and the choice of encapsulation materials such as chitosan, alginate and protein polymers to achieve synchronised peptide‐polysaccharide release with gut transit and local immune tissue activation for proper mucosal and systemic innate response activation (Dezfooli et al. 2019; Giri et al. 2021). The various nano‐formulations produce different release patterns that start with immediate non‐specific defence enhancement through rapid bursts and then provide extended release for sustained immune surveillance to maximise phagocyte activation, lysozyme activity and immune gene expression (Giri et al. 2021; Luis et al. 2017). The controlled release mechanism extends drug presence in the body, which reduces the need for frequent dosing and prevents peak and trough fluctuations that lead to tolerance development and waste production, thus reducing feed costs while maintaining pathogen protection through continuous mucosal stimulation (Dezfouli et al. 2019; Giri et al. 2021; Luis et al. 2017). Nano‐encapsulation technology controls the release pattern to achieve optimal immunostimulation timing between immediate and prolonged effects, which results in better performance and lower feed consumption in aquaculture (Dezfouli et al. 2019; Giri et al. 2021; Luis et al. 2017).

### 5.2 Enhanced Bioavailability and Bioactivity

**FIGURE 7:**
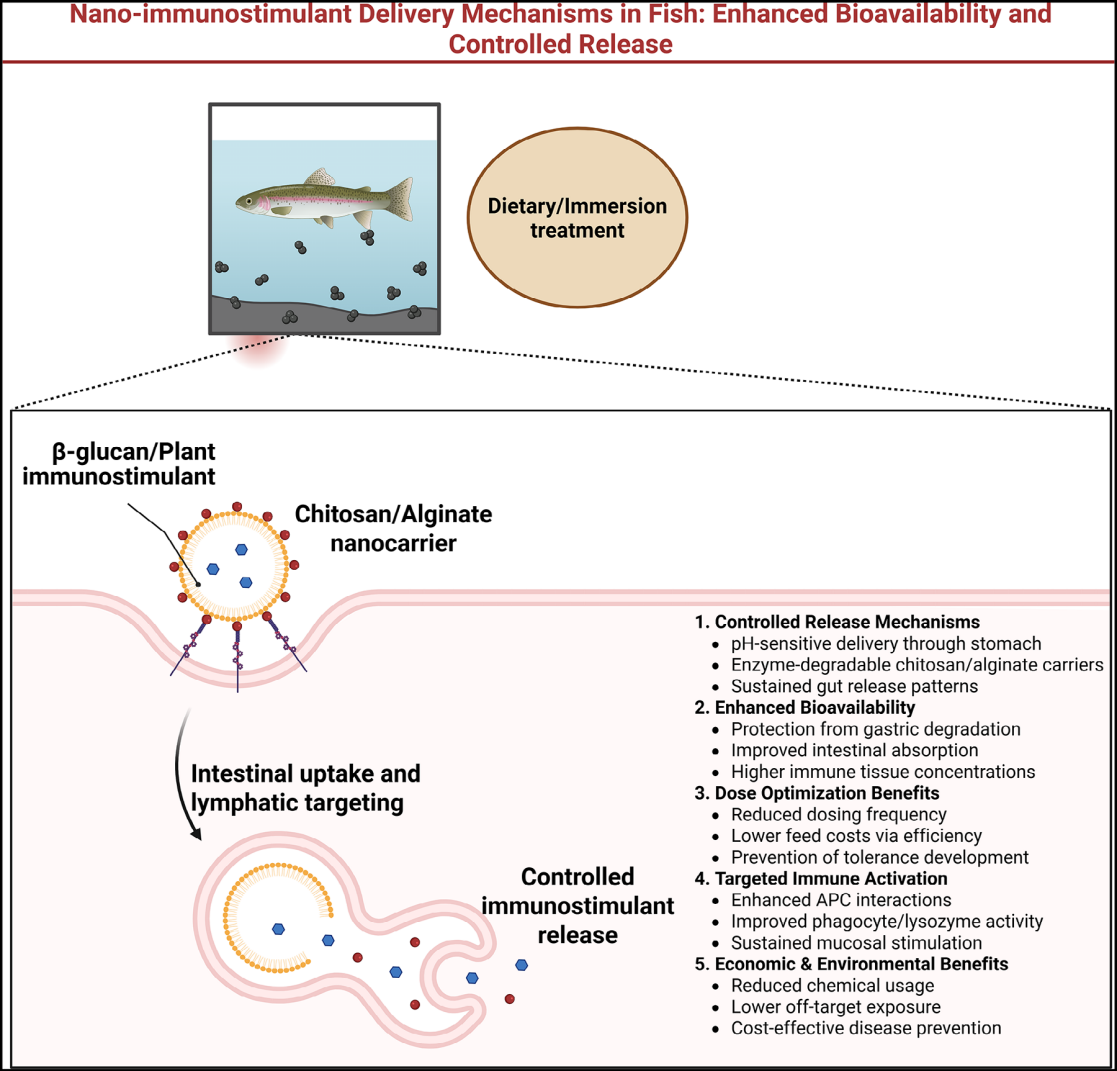
Conceptual diagram detailing the mechanical pathways of enhanced bioavailability, controlled release and systemic targeting in nano‐formulations.

Nano‐formulations enhance the bioavailability of poorly soluble immunostimulants through three mechanisms: solubility enhancement and protection against gastric degradation and improved mucosal absorption in the gut. The intestinal environment allows poorly soluble active compounds to dissolve from biocompatible carriers, including chitosan and alginate, and lipid‐based systems, which results in higher local active concentrations at immune tissue sites (Harshitha et al. 2023). Nanocarriers enhance drug pharmacokinetics by delivering medications to lymphatic tissues and gut‐associated lymphoid tissues, which results in better activation of phagocytic and mucosal immune systems and less drug loss in the body (Ruyra et al. 2013; Thwaite et al. 2018). The release pattern of these systems can be engineered to produce either immediate burst delivery or prolonged sustained release profiles that match the activation periods of immune cells (Ruyra et al. 2013; Thwaite et al. 2018). The pharmacokinetic benefits of this method include reduced clearance rates, longer gut residence time and improved immune effector site delivery, which could minimise the amount of medication needed (Ruyra et al. 2013; Z. Zhang et al. 2019). The encapsulation method protects sensitive compounds from enzymatic and pH‐related breakdown during digestion because it creates a protective barrier that allows controlled release at specific target locations, which enhances stability and functional performance in fish feeds (Harshitha et al. 2023; Z. Zhang et al. 2019). Nanocarriers enable better drug delivery, which results in enhanced treatment outcomes, extended drug action and sustained immune system activation (Ruyra et al. 2013; Thwaite et al. 2018; Z. Zhang et al. 2019). The mechanisms of enhanced bioavailability and controlled release in NIS delivery systems for fish are shown in Figure 7.

### 5.3 Dose Optimisation and Efficacy Improvement

NISs produce therapeutic effects at reduced doses because they deliver improved targeted delivery to APCs and controlled release mechanisms that boost local immune activation and bioavailability while reducing the amount of stimulus needed for effective responses (Sun et al. 2017). The nanoscale delivery system enables immunostimulants to reach immune contact points where they enhance APC interaction and drug release management, which results in lower drug consumption and either preserved or improved therapeutic effects (Cornet et al. 2021; Sun et al. 2017). Research shows that nano‐formulations in fish produce strong innate immune responses through liposome and NP carriers, which require lower concentrations than free agents, while specific optimisation needs exist based on species and formulation type, as demonstrated by poly I:C and LPS‐loaded liposomes and comparable nanocarriers (Ruyra et al. 2013). NISs offer economic and environmental benefits because they require smaller doses than conventional immunostimulants, which have poor absorption and fast degradation properties (Abd Allah et al. 2023). The implementation of dose optimisation techniques leads to cost reduction and environmental benefits because it uses fewer active ingredients and reduces off‐target exposure while delivering precise delivery systems that boost bioavailability according to NISs and β‐glucan/plant‐based immunostimulants in aquaculture (Ching et al. 2020; Meena et al. 2013). The use of reduced dosing practices in fish farming results in environmental and economic advantages because it decreases chemical use and enhances disease resistance according to various reviews and experimental studies on different fish species (Messina et al. 2023; Sanchez et al. 2017). Nevertheless, dose sparing should not be assumed for all formulations. Some studies report clear immune enhancement only within a narrow concentration range, whereas higher doses may induce oxidative stress, inflammation, reduced growth, or tissue damage, particularly for metallic NPs. Future studies should therefore report full dose–response curves, no‐observed‐adverse‐effect levels and challenge‐based survival outcomes rather than relying only on immune biomarker changes. To prevent overgeneralisation and maintain scientific rigour, quantitative improvements in fish immune performance and disease resistance must be strictly contextualised within their specific experimental frameworks. For instance, the documented increases in relative percent survival (RPS), which typically range from 20% to over 80% against aggressive pathogens such as *A. hydrophila*, *Vibrio parahaemolyticus* or *Yersinia ruckeri*, are inherently bound to specific host species (such as Nile tilapia, rainbow trout, or white leg shrimp), defined life stages, and highly controlled laboratory challenge models. These numerical values represent optimised maximum efficacies achieved under stable, low‐stress environments with minimal biological variables. Consequently, direct extrapolation of these quantitative metrics to commercial production scales introduces substantial uncertainty. In large‐scale aquaculture ponds, cages, or intensive systems, dynamic environmental stressors, including fluctuating dissolved oxygen, variable water temperatures, high organic matter loads and complex multi‐pathogen pressures, can fundamentally alter NP stability, gut transit times and mucosal uptake efficiency, potentially dampening the high protection percentages observed in laboratory trials.

### 5.4 Stability and Storage Advantages

Nano‐formulations enhance the storage stability and shelf life of immunostimulants in fish feed by protecting active ingredients within robust nanocarriers, reducing degradation pathways and enabling controlled release during storage and after ingestion (Fitria et al. 2021; Ruyra et al. 2013; Xue et al. 2021). The encapsulation of immunostimulants in liposomes and polymeric NPs and Pickering emulsions protects these compounds from hydrolysis and enzymatic degradation and oxidation and maintains their bioactivity until they reach immune interfaces for release (Ruyra et al. 2013; Xue et al. 2021). Nanocarriers enhance the solubility and dispersion properties of feed matrices, which prevents phase separation and sedimentation and reduces potency stability over time (Y. Lu et al. 2024). The storage conditions determine shelf‐life benefits because encapsulated formulations maintain their thermal and oxidative stability, while pro‐oxidants cannot penetrate, and the particles stay intact in terms of size and zeta potential at room temperature and refrigeration (Esmaili et al. 2018; Xue et al. 2021; Zhao et al. 2018). Nano‐emulsions and nano‐encapsulated systems protect bioactive compounds from food system degradation by providing them with enhanced stability and activity and better resistance to hydrolytic and oxidative breakdown than free agents (Bilbao‐Sáinz et al. 2012; Zhao et al. 2018). The mentioned characteristics enable lower medication amounts to be used over time, which decreases expenses and environmental impact while maintaining both effectiveness and fish health results (Fitria et al. 2021; Sun et al. 2017; Xue et al. 2021).

### 5.5 Environmental Impact: Potential Reduction and Remaining Risks

NISs may reduce environmental loading when they improve bioavailability, lower the required dose of active compounds and decrease off‐target release. However, this benefit is formulation‐dependent and should not be generalised across all NP classes. Biodegradable polymeric and lipid‐based systems may offer lower persistence than many inorganic materials, whereas metallic and carbon‐based NPs may accumulate in sediments, interact with DOM, or affect non‐target organisms. Therefore, environmental benefit should be demonstrated through fate modelling, predicted environmental concentration (PEC) assessments and multispecies ecotoxicity testing rather than being assumed from nanoscale delivery alone (Kah et al. 2016; Kookana et al. 2014). Nano‐formulations allow targeted delivery and extended residence at infection sites, which enables the use of smaller active ingredient amounts to achieve the same effect as traditional agrochemicals, thus reducing overall environmental release (Awad et al. 2022; Kookana et al. 2014). The environmental behaviour of these substances depends on their formulation and size and surface characteristics, which determine their ability to sorb and degrade under light and their movement through the environment; multiple studies show that nano‐formulations breakdown more quickly than bulk artificial intelligence (AI) with established degradation patterns that decrease the chance of water pollution (Kah et al. 2018; Praetorius et al. 2012). The assessment of risks depends on exact fate modelling and PEC calculations for engineered NPs to establish lower exposure scenarios during NIS design and testing (O'Brien and Cummins 2010). The combination of reduced medication amounts and precise delivery methods helps decrease environmental impact by reducing water pollution and lowering the concentration of effluent in water bodies, which protects both target and non‐target species and their habitats (Garner and Keller 2014; Kookana et al. 2014). The breakdown of residues occurs through photolysis and microbial processes, which environmental factors trigger to reduce their persistence in water bodies and soil compared to conventional agents (Kah et al. 2018; Sun et al. 2014).

### 5.6 Cost‐Effectiveness Analysis

The economic value of NISs depends on whether improved health outcomes compensate for formulation, manufacturing, quality control, storage and regulatory costs. Potential benefits include reduced disease losses, improved survival, better feed conversion, lower antibiotic use and fewer treatment interventions. However, cost‐effectiveness remains difficult to generalise because most studies report immunological or experimental challenge outcomes rather than farm‐scale economic indicators. Commercial feasibility therefore requires data on production cost per kilogramme of treated feed, shelf life under storage, dose frequency, survival improvement under field conditions and return on investment in semi‐intensive and intensive systems (Bulfon et al. 2013; Narsale et al. 2024). Research shows that immunostimulants such as β‐glucans and polysaccharides and microalgal/nanostructured derivatives enhance innate immunity, which leads to decreased disease incidence, improved feed conversion efficiency, reduced total production losses and elevated survival rates (Khanjani et al. 2022; Ma et al. 2020; W. Wang et al. 2016). The initial expenses for adoption (dosing equipment and quality control and monitoring tools) can be compensated through long‐term health benefits, reduced antibiotic use and enhanced market results, which create positive returns in semi‐ to intensive systems (Asiri and Chu 2020; Bulfon et al. 2013). Cost‐effectiveness hinges on factors such as (i) ingredient and production costs, (ii) measured efficacy in target species/diseases, (iii) reductions in mortality and feed waste, (iv) labour and management requirements and (v) policy and financing support for adoption (Karimi et al. 2018; Tsani and Koundouri 2018). The combination of initial expenses and long‐term health benefits and production advantages leads to economic benefits (Ching et al. 2020; Khanjani et al. 2022; Parker et al. 2020; W. Wang et al. 2016). As shown in Table 4, biodegradable polymeric systems, particularly chitosan‐, alginate‐ and PLGA‐based carriers, currently appear to have the highest translational potential for feed‐based or mucosal immunostimulation because they combine payload protection, biodegradability and comparatively favourable safety profiles. Lipid‐based platforms are also promising, especially for hydrophobic or nucleic acid‐based immunostimulants, but require further optimisation for storage stability and performance under variable aquaculture conditions. In contrast, metallic‐ and carbon‐based nanomaterials may offer strong antimicrobial or carrier functions, but their routine use is constrained by narrower safety margins, environmental persistence, bioaccumulation potential and limited long‐term field data.

**TABLE 4:** Critical decision matrix for nano‐immunostimulant platforms in aquaculture.

Nanoparticle platform	Main application potential	Efficacy evidence	Key advantages	Main limitations	Safety/environmental concern	Scalability/commercial readiness	Overall translational priority	References
Chitosan‐based nanoparticles	Oral immunostimulants, mucosal vaccines, feed additives	Moderate to strong experimental evidence in fish models	Mucoadhesive, biodegradable, improves intestinal retention, protects antigens and bioactives	Variable polymer quality, pH‐dependent behaviour and feed‐processing stability	Generally, favourable, but dose, molecular weight and formulation must be validated	Relatively high; compatible with feed‐based delivery	High	Chua et al. (2011); Dawood et al. (2020); Gao et al. (2016); Guadarrama‐Escobar et al. (2023); Y. Liu et al. (2021); Madrid et al. (2021); Rosidah and Mulyani (2022)
Alginate/chitosan hybrid systems	Oral delivery of proteins, antigens, plant extracts and probiotic‐derived compounds	Moderate evidence; often formulation‐dependent	Gastric protection, pH‐responsive release, biocompatibility and improved encapsulation	Encapsulation efficiency and release kinetics can vary	Low to moderate concern when biodegradable materials are used	Moderate to high; requires standardisation	High	Dixit et al. (2014); Fitriagustiani et al. (2022); Gao et al. (2016); Verma et al. (2018)
PLGA‐based polymeric carriers	Vaccine/adjuvant delivery and sustained antigen release	Moderate evidence; stronger in biomedical delivery literature than aquaculture‐specific studies	Controlled release, antigen protection, APC uptake and tuneable degradation	Higher cost, formulation complexity and limited aquaculture field data	Generally, biodegradable, but degradation products, dose and clearance require evaluation	Moderate; cost and manufacturing complexity remain barriers	Moderate to high	Dixit et al. (2014); Guo et al. (2019); Hogarth et al. (2021); Nicolete et al. (2011); Vivek et al. (2014)
Liposomes/nanoliposomes	Delivery of LPS, dsRNA, hydrophobic immunostimulants and vaccines	Moderate experimental evidence, including fish innate immune stimulation	Biocompatible, suitable for hydrophobic or nucleic acid payloads and useful for mucosal delivery	Storage instability, oxidation, leakage and lipid/surfactant optimisation	Usually, favourable, but depends on lipid composition, surfactants and additives	Moderate; stability and cost issues remain	Moderate to high	S. Ahmed et al. (2011); Guo et al. (2019); Hogarth et al. (2021); Ji et al. (2015); Lehn et al. (2014)
Nanoemulsions/solid lipid nanoparticles	Hydrophobic plant extracts, essential oils and nutraceutical immunostimulants	Emerging to moderate evidence	Improves solubility, dispersion, palatability and bioavailability of hydrophobic compounds	Phase separation, Ostwald ripening, salinity sensitivity	Low to moderate; depends on surfactant and oil phase	Moderate; promising for feed additives	Moderate	Hogarth et al. (2021); Ji et al. (2015); Lehn et al. (2014)
Selenium nanoparticles	Trace‐element immunomodulation, antioxidant support and disease resistance	Moderate evidence across several fish species	Supports antioxidant defence and immune responses at optimised doses	Narrow dose window; excess selenium can be toxic	Bioaccumulation, dietary residue and dose‐related toxicity require monitoring	Moderate; feasible but dose‐sensitive	Moderate	Dezfouli et al. (2019); Saad et al. (2022)
Zinc oxide nanoparticles	Antimicrobial support, immune‐gene modulation and disease‐resistance enhancement	Moderate evidence, but outcomes are variable	Antimicrobial activity, trace‐element function and immune modulation	Dose‐dependent oxidative stress, developmental toxicity and inflammatory effects	Tissue accumulation and ecotoxicity concerns require careful assessment	Moderate; regulatory caution needed	Moderate to low	Choi et al. (2016); Handy et al. (2008); Kaur et al. (2019); Ribeiro et al. (2023)
Silver nanoparticles	Antimicrobial applications and pathogen control	Strong antimicrobial evidence; immunostimulant evidence is less consistent	Broad antimicrobial activity	Narrow safety margin, cytotoxicity, gill toxicity and embryotoxicity	High concern due to persistence, bioaccumulation and non‐target toxicity	Low for routine feed use; possible restricted use in disinfection or pathogen‐control contexts	Low	George et al. (2012); Handy et al. (2008); Luis et al. (2021); Shaalan et al. (2017)
Carbon nanotubes/graphene‐based materials	Experimental vaccine/adjuvant carriers and high‐loading platforms	Limited aquaculture‐specific evidence	High surface area, functionalisation capacity and payload loading	Limited safety data, aggregation, possible chronic toxicity	High uncertainty regarding persistence, trophic transfer and long‐term toxicity	Low; mainly experimental	Low	Y. Liu et al. (2013); Marchesan et al. (2015); Orecchioni et al. (2014); S. P. B. Sousa et al. (2020)

Although current peer‐reviewed studies support the biological benefits of NISs and nano‐enhanced feeds, direct farm‐level ROI or benefit–cost comparisons with conventional antibiotic treatments remain scarce. Available studies mainly report improved growth, feed conversion, survival, immune response and disease resistance rather than explicit net profit or ROI calculations (Dube 2024; S. K. Khan et al. 2024; Okeke et al. 2022). Therefore, ROI can presently be discussed only as an illustrative model based on reported biological outcomes. For example, in a semi‐intensive farm producing 10 tons of fish, a disease outbreak causing 20% mortality would result in approximately 2 tons of lost biomass. At a market price of $3 per kg, this equals an estimated gross loss of $6000. If a dietary NIS program costing $400–$800 per production cycle reduces mortality by 30%–50% relative to an untreated or antibiotic‐dependent scenario, approximately 600–1000 kg of biomass losses could be avoided, equivalent to $1800–$3000 in retained production value. Under this simplified assumption, the estimated return may be approximately 2.25‐ to 3.75‐fold, excluding additional benefits such as improved feed conversion ratio (FCR), reduced antibiotic expenditure, fewer withdrawal‐period losses, lower residue‐related market risks and improved consumer acceptance. Thus, while NISs may involve higher initial formulation costs than conventional treatments, their preventive function may generate favourable economic outcomes when biological improvements are translated into avoided mortality and improved production efficiency. However, future studies should include standardised bioeconomic models, net profit, benefit–cost ratio and ROI comparisons to validate these assumptions under commercial farming conditions (Nasr‐Eldahan et al. 2021; Nolasco‐Alzaga et al. 2025; Seethalakshmi et al. 2021).

### 5.7 Critical Synthesis, Contradictory Evidence and Limitations of Current Evidence

A critical evaluation of the current literature reveals that while the biological potential of NISs is immense, the field is constrained by significant methodological heterogeneity and contradictory findings. The strength and quality of current evidence vary dramatically across NP platforms. Polymeric and lipid‐based nanocarriers possess the most robust data regarding mucosal safety and structural biocompatibility; however, their performance remains highly unpredictable under varying environmental salinities and high‐temperature feed processing conditions, often leading to premature payload leakage or polymer relaxation.

In contrast, metallic and metal‐oxide NPs (e.g., AgNPs, ZnONPs, SeNPs) exhibit the sharpest contradictions in published data. For instance, while several studies report marked upregulation of immune genes and elevated survival rates in Nile tilapia exposed to dietary ZnONPs or SeNPs, parallel toxicological studies at identical concentrations frequently report severe oxidative stress, lipid peroxidation and histopathological lesions in the liver and gills. These striking contradictions stem from a widespread failure in the literature to standardise NP surface coatings, dissolution rates and exact particle sizes, which fundamentally dictate whether a metallic particle acts as a beneficial micronutrient stimulant or a severe cellular toxin.

Furthermore, a systemic limitation of the current body of research is its heavy reliance on short‐term, tightly controlled laboratory challenge models. The vast majority of peer‐reviewed trials assess immune biomarkers for only a few weeks following an acute pathogen injection. Consequently, there is an absolute scarcity of long‐term evidence regarding chronic tissue accumulation, tolerance development, intestinal microbiome shifts and multigenerational ecotoxicity. Most importantly, the translational quality of the current evidence remains low because very few studies validate these nano‐enhanced feeds under real‐world, field‐scale aquaculture conditions where dynamic stressors such as fluctuating dissolved oxygen, variable organic matter and complex multipathogen exposure coexist. Until standardised eco‐toxicological guidelines and commercial‐scale trials are established, extrapolating laboratory‐scale biomarker enhancements to predict net commercial profitability remains a significant scientific challenge.

## 6 Safety Assessment and Toxicological Considerations

Safety findings for NISs are not always consistent across studies because toxicity depends on particle composition, size, coating, dissolution rate, dose, exposure duration, water chemistry and fish life stage. For example, trace‐element NPs such as Se or ZnO may improve antioxidant and immune responses at optimised dietary levels but can induce oxidative stress, altered enzyme activity, developmental effects, or tissue damage at higher or prolonged exposures. Similarly, AgNPs may reduce pathogen loads but can also cause gill injury, embryotoxicity, cytotoxicity, or bioaccumulation. These contradictions highlight the importance of reporting dose–response relationships, particle characterisation, exposure route and no‐effect thresholds in future studies.

### 6.1 NP Accumulation and Bioaccumulation

Research on NISs in fish is developing, but existing studies on nanomaterial bioaccumulation and fish tissue distribution patterns indicate that scientists can evaluate tissue distribution and accumulation factors and clearance mechanisms. NISs spread through tissues based on lipid content and their ability to accumulate in specific organs. The liver and gill tissues accumulate the highest amounts of NISs because they serve as metabolic centres and filtration organs, which results in accumulation patterns that follow typical NP behaviour (Arnot et al. 2010; Y. Zheng and Nowack 2022). The accumulation of NPs depends on their size and surface properties because their small dimensions and specific surface modifications lead to better tissue penetration and cellular uptake, but hydrophobic and lipophilic coatings increase BAF/BCF values in lipid‐rich tissues (Arnot et al. 2010; Y. Zheng and Nowack 2022). The body responds differently to NPs based on their chemical makeup because metal‐based and carbon‐based NPs follow separate paths of distribution and elimination in the body, with hydrophobic NPs remaining in tissues longer than hydrophilic NPs (Arnot et al. 2010; Y. Zheng and Nowack 2022). The body eliminates substances through biliary excretion and renal filtration and macrophage‐mediated clearance, but tissue elimination half‐lives depend on both tissue characteristics and exposure pathways. Toxicology studies show that substances remain in tissues longer when exposed to substances for long periods because higher trophic level tissues such as the liver hold onto substances (Fisk et al. 2001; Muir et al. 2017; Ogorek et al. 2021). The field requires full compound‐specific information about NISs, yet current research shows that they follow established bioconcentration principles (Arnot et al. 2010; Y. Zheng and Nowack 2022). For metal‐based NPs, the potential persistence of residues in edible tissues also highlights the need to define appropriate withdrawal periods before harvest. Unlike conventional therapeutics, standardised withdrawal times for many NP‐based immunostimulants are not yet established and may vary depending on particle composition, dose, exposure duration, tissue accumulation and clearance rate. Therefore, residue monitoring in muscle and major accumulation organs, such as the liver and gills, should be considered when evaluating the food safety and commercialisation potential of metal NP applications in aquaculture.

### 6.2 Tissue Distribution and Clearance Mechanisms

**FIGURE 8:**
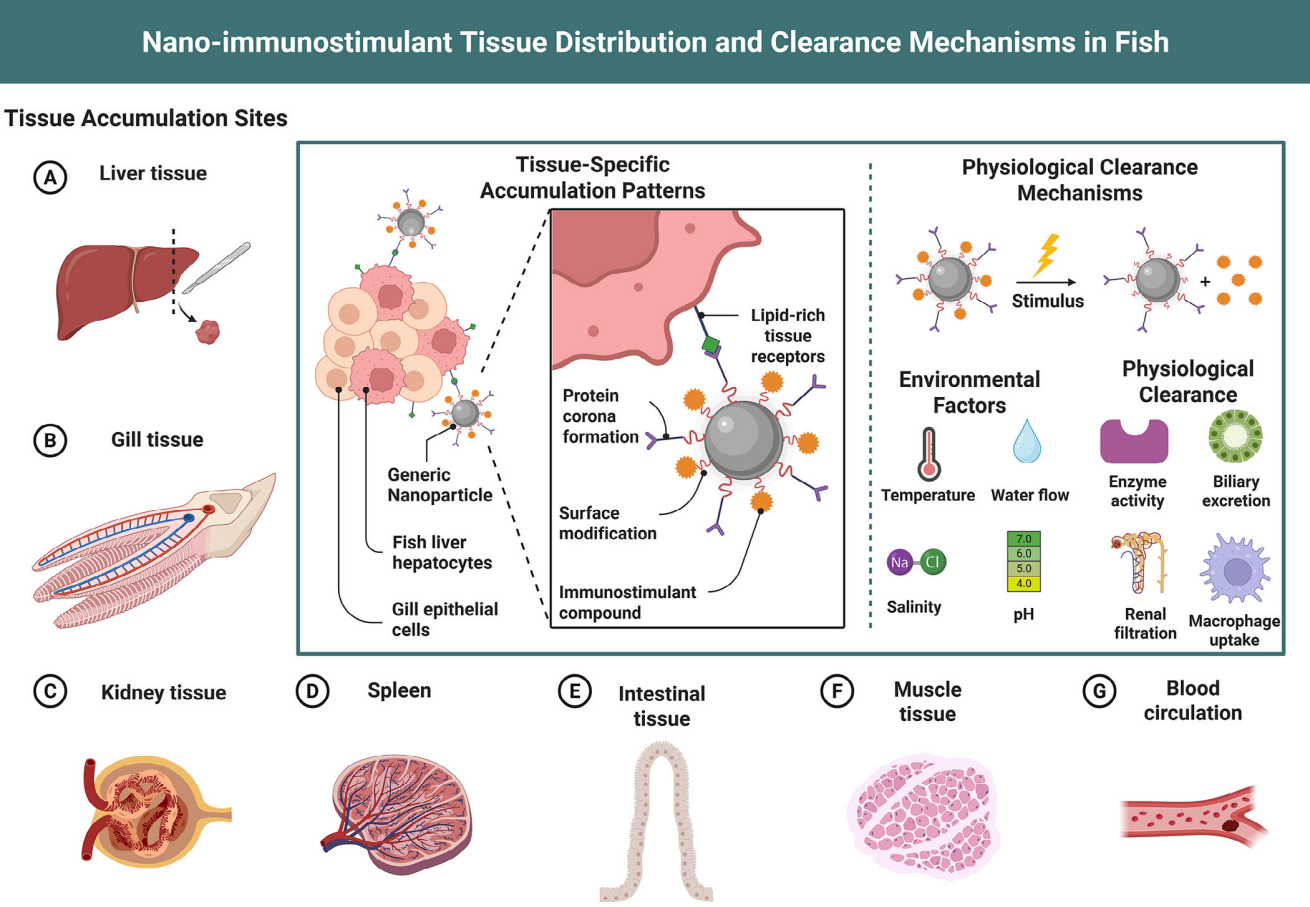
Evidence‐based summary of tissue‐specific distribution patterns, bioaccumulation sinks and metabolic clearance pathways of nanoparticles in fish.

- NISs are distributed unevenly throughout fish bodies because they primarily accumulate in liver and gill tissues, which serve as the first point of contact for metabolite processing and substance filtration, but other organs take up these substances at varying concentrations depending on exposure techniques and NP properties (Cedervall et al. 2012; Kashiwada 2006; Saad et al. 2022). The size of NPs together with their surface chemistry and material composition determines how they distribute within tissues: Small particles with hydrophobic coatings tend to penetrate lipid‐rich tissues while entering cells, but they remain in fatty tissues longer. The organ distribution and clearance behaviour of metal‐ and carbon‐based nanomaterials differ from each other (Kashiwada 2006; Xu et al. 2018). The structures of the protein corona that form on NPs determine their transport behaviour and fat metabolism, which results in modified tissue distribution patterns that regulate metabolic and immune system functions (Cedervall et al. 2012).
- The body eliminates substances through three main processes: biliary excretion and renal filtration and macrophage/MPS‐mediated sequestration for elimination. The rate of elimination through hepatobiliary routes depends on tissue type, but renal routes provide faster clearance for small hydrophilic particles (Xu et al. 2018). The elimination rate depends on endocytic uptake at gills and intestinal epithelia followed by intracellular processing and biotransformation, which affects clearance rates through chemical form and organismal factors (James and Kleinow 2014; Kashiwada 2006).
- The distribution patterns of vaccines affect safety because they lead to the accumulation of potential irritants in detoxification organs such as the liver and kidneys, and they impact vaccine efficacy by directing the vaccine to antigen‐presenting tissues and immune organs such as the spleen and hindgut to boost immunogenicity. The prolonged tissue retention and reduced elimination rate of vaccines increase the risk of long‐term exposure, but it enhances vaccine effectiveness when the vaccine targets areas with high numbers of APCs (Harshitha et al. 2023; C. Zhang et al. 2020). Figure 8 illustrates the tissue distribution patterns and clearance mechanisms of NISs in fish, highlighting the primary accumulation sites and factors affecting elimination rates.

### 6.3 Cytotoxicity and Genotoxicity Assessment

There is no single official standardised protocol for NISs in fish; however, validated approaches converge on in vitro and ex vivo cytotoxicity and genotoxicity testing in fish cell lines and primary cultures using OECD‐validated assays (Connolly et al. 2015; Giri et al. 2018). The assessment of cytotoxicity through standardised methods involves cell viability measurements and membrane integrity tests and oxidative stress marker evaluations using RTL‐W1 and RTG‐2 rainbow trout cell lines based on comparative cytotoxicity research (Connolly et al. 2015). The comet assay and micronucleus test have been modified for fish cells to assess DNA damage from NISs according to studies on immunomodulatory NPs in teleost models (Connolly et al. 2015; Torrealba et al. 2016). The three main mechanistic pathways of toxicity include oxidative stress and lysosomal disruption and inflammatory cytokine regulation. The implementation of dose optimisation techniques together with surface coating methods and delivery system designs such as liposomal‐ and protein‐based nanostructures helps minimise cytotoxic effects while maintaining immunostimulatory properties (Ruyra et al. 2013; Torrealba et al. 2016). The determination of non‐toxic fish immunostimulant concentrations requires EC_50_ benchmarks, but researchers need to confirm cell types and test exposure periods according to established fish immunostimulant paradigms to prevent adverse effects (Abarike et al. 2019; Yue et al. 2017).

### 6.4 Environmental Fate and Ecotoxicology

NISs undergo environmental changes in aquatic systems through processes that depend on sunlight exposure, DOM, pH levels, ionic strength and microbial and chemical reactions. The stability, bioavailability and toxicity of non‐target organisms change when TiO_2_‐ and Ag‐based nanomaterials experience photochemical and sulphidation and dissolution and oxidative transformations (Levard et al. 2012; Luo et al. 2020; Sun et al. 2014). The duration substances stay in water depends on natural organic matter (NOM) and DOM because these substances either enhance or reduce the rate of aggregation and disaggregation processes (Lv et al. 2017; Yin et al. 2015). The process of photo‐irradiation causes TiO_2_ to age, which results in modified surface characteristics that affect its interactions with biological organisms (Sun et al. 2014). The bioavailability and toxicity of metals such as Ag and Cu to phytoplankton and invertebrates are affected by metal ageing in metals and sulphidation processes in sediments (J.‐Y. Liu et al. 2011; Vignardi et al. 2023). The accumulation of sediment over time together with occasional disturbance events leads to increased exposure of benthic and pelagic organisms to contaminated sediments, which can modify ecosystem functions by changing community structure and nutrient distribution patterns (Binh et al. 2014; Harrison et al. 2023). The assessment of risk depends on three essential components: environmental transformation prediction and seasonal and ageing factor integration and multispecies ecotoxicity endpoint evaluation for water column and sediment compartments (L. Fang et al. 2011; Harrison et al. 2023). The tissue‐specific accumulation patterns and toxicological thresholds for both commercial fish and non‐target aquatic organisms are systematically summarised in Table 5. In addition to transformation processes, the ecological risks of NISs to non‐target organisms surrounding aquaculture ponds should be considered. Residual NPs released through uneaten feed, faeces or pond effluents may interact with primary producers and lower trophic organisms such as microalgae, cyanobacteria, *Daphnia* spp., copepods, benthic invertebrates and sediment‐associated microbial communities. Metal‐based NPs, particularly Ag‐, Cu‐, ZnO‐ and TiO_2_‐based materials, may inhibit algal growth, reduce photosynthetic efficiency, induce oxidative stress, alter membrane integrity and impair reproduction or survival in zooplankton and invertebrates, depending on particle size, concentration, dissolution rate, surface coating and water chemistry. Such effects may indirectly disturb pond food webs by reducing primary productivity, modifying grazing pressure and altering nutrient cycling. Therefore, environmental risk assessment of NISs should include chronic and sublethal endpoints in non‐target organisms, including algal growth inhibition, *Daphnia* immobilisation or reproduction assays, benthic toxicity tests and microbial community analyses under realistic pond water and sediment conditions.

**TABLE 5:** Toxicological profiles and bioaccumulation patterns of metallic nanoparticles in aquaculture systems.

Nanoparticle type	Target fish species	Main bioaccumulation sites (fish)	Representative LC_50_ values (exposure medium, duration)	Impacts on non‐target/other aquatic organisms	References
*Silver NPs (AgNPs)*	Siberian sturgeon (*Acipenser baerii*) larvae	Not quantified, but histology shows damage in *epidermis, gills, liver* indicating target tissues [epidermis and gill epithelia, hepatic tissue]	96 h LC_50_ ≈ 15.0 ± 2.9 mg/L (waterborne AgNPs)	Same study reports higher toxicity of CuNPs than AgNPs to sturgeon, but no invertebrates tested	Ostaszewska et al. (2016)
*Silver NPs (AgNPs)*	Common carp (*Cyprinus carpio*)	*Liver > intestine > gills > muscle* for blood‐mediated AgNPs; similar pattern (gills, intestine, liver, muscle) for plant‐ and onion‐peel‐mediated AgNPs	Blood‐mediated AgNPs: sublethal range 0.03–0.09 mg/L (long‐term); 96 h LC_50_ not directly reported but mortality and histological damage increase at 0.09 mg/L. Green AgNPs (*Allium cepa*): 96 h LC_50_ ≈ 6–10 mg/L (biogenic AgNPs; reported as 2–10 mg/L test range with LC_50_ derived within)	No direct non‐fish test in same experiments; broader work shows AgNPs are highly toxic to *Daphnia* and algae with LC_50_ in low‐µg/L–mg/L range	Haghighat et al. (2021); Kakakhel et al. (2021); Q. Khan et al. (2025); Krishnasamy Sekar et al. (2023)
*Silver NPs (AgNPs)*	Zebrafish (*Danio rerio*), adults	Primarily *gills and liver*; brain can also accumulate Ag in some acute tests	Biogenic AgNPs: 96 h LC_50_ ≈ 142 µg/L (*Malva crispa*‐mediated AgNPs); biologically synthesised AgNPs vs. AuNPs: 96 h LC_50_ ≈ 24.5 µg/L for AgNPs (much lower than 41 mg/L for AuNPs)	Acute OECD tests show AgNPs are ‘category acute 2’ for fish (*Oryzias latipes*; LC_50_ tens of µg/L) but ‘acute 1’ for *Daphnia* and some algae, indicating higher sensitivity of invertebrates and primary producers	Kovrižnych et al. (2013); Krishnaraj et al. (2016); Ramachandran et al. (2018); Sohn et al. (2015)
*Silver NPs (AgNPs)*	Rainbow trout (*Oncorhynchus mykiss*)	*Gills and liver* are primary accumulation sites; some studies also detect Ag in kidney	Wastewater‐borne AgNPs: chronic 28‐day exposure 1.4–36.2 µg/L with measurable accumulation but no growth effect; acute LC_50_ for colloidal AgNPs in trout ≈ 2.16 mg/L; other trout studies use 40 µg/L AgNPs and 4 µg/L Ag^+^ without direct LC_50_ but show clear sublethal toxicity	AgNPs in trout elicit immunotoxicity (reduced phagocytosis, altered cyclooxygenase activity) and oxidative stress; OECD tests show *Daphnia pulex* LC_50_ as low as 40 µg/L for nanosilver, indicating higher non‐fish sensitivity	Bruneau et al. (2016); Griffitt et al. (2008); Hernández‐Moreno et al. (2024); Shaw et al. (2012); Zeumer et al. (2020)
*Silver NPs (AgNPs)*	Nile tilapia (*Oreochromis niloticus*), *Tilapia zillii*	Brain examined; systemic distribution expected but not fully mapped	96 h LC_50_ ≈ 19.5 ± 2 mg/L (*O. niloticus*) and 20 ± 2.4 mg/L (*T. zillii*)	Same lab reports ZnONPs 96 h LC_50_ in *O. niloticus* ≈3.1 ± 0.4 mg/L, indicating ZnONPs may be more acutely toxic than AgNPs to this fish; no parallel invertebrate testing in this paper	Afifi et al. (2016)
*Silver NPs (AgNPs)*	Goodeid fish *Chapalichthys pardalis*	Liver and gills show strongest oxidative damage; muscles also affected	96 h LC_50_ ≈ 10.3 mg/L (waterborne AgNPs)	Non‐fish species not included; but AgNPs widely reported to be more toxic to *Daphnia* and some algae than to fish, with LC_50_ down to tens of µg/L	Griffitt et al. (2008); Valerio‐García et al. (2017)
*Silver NPs + ZnONPs (mixtures)*	Zebrafish (*D. rerio*), diet and water exposure	For mixtures, *liver and intestine* are major Ag/Zn sinks; gills also accumulate Ag, especially with ZnO co‐exposure	Dietary AgNPs: 10–50 mg Ag/kg feed; ZnONPs: 100 mg ZnO/kg feed for 28 days (no LC_50_ because diet‐based). Waterborne acute: co‐exposure changes AgNP 96 h LC_50_ (co‐exposed fish show reduced acute mortality but enhanced chronic tissue damage)	Non‐fish: separate experiments show tadpoles (*Polypedates maculatus*) are highly sensitive to Ag/ZnONPs with significant haematological and oxidative stress effects at 1–50 mg/L	Mahjoubian et al. (2023); Moghadam et al. (2025); Murthy et al. (2022); Sibiya et al. (2024)
*Zinc oxide NPs (ZnONPs)*	Mozambique tilapia (*Oreochromis mossambicus*)	Mainly *liver*, but systemic oxidative stress markers measured in gill, liver and serum	Waterborne ZnONPs: 96 h LC_50_ not explicitly given, but acute tests used 0.05–0.20 mg/L; chronic 21‐day sublethal exposures at fractions of these doses; effects evident well below mg/L. For *O. niloticus*, ZnONP 96 h LC_50_ ≈ 3.1 mg/L (same lab as AgNP brain study)	Same tilapia study also evaluated AgNPs and combined Ag/ZnONPs; chronic mixture exposure produced more severe gill and liver lesions than individual NPs, implying potential risk to other aquatic species at similar levels	Afifi et al. (2016); Sibiya et al. (2024)
*Zinc oxide NPs (ZnONPs)*	Zebrafish (*D. rerio*)	Mainly *gills, liver, intestine* (from zebrafish NP reviews summarising multiple ZnO studies)	Various zebrafish studies report ZnONP LC_50_ in mg/L range, often 1–5 mg/L for acute waterborne exposure, although exact values differ by coating, size and medium	Reviews highlight higher sensitivity of invertebrates (*Daphnia*) and algae to ZnONPs (growth inhibition and immobilisation at low‐mg/L and sub‐mg/L levels) compared to fish	Chakraborty et al. (2016), d'Amora et al. (2022), Griffitt et al. (2008), Shaw and Handy (2011)
*Selenium NPs (SeNPs)*	(Limited direct fish LC_50_ data in this set; mainly captured in broader metallic NP reviews)	Reviews note preferential accumulation in *liver and kidney*, consistent with ionic/bulk Se behaviour, but robust SeNP‐specific fish bioaccumulation maps are still scarce	Specific SeNP LC_50_ values in fish are not reported in these retrieved abstracts; most SeNP data in fish are sublethal or mechanistic (oxidative stress, endocrine effects) rather than formal LC_50_ testing	Several reviews emphasise that SeNPs can be toxic to aquatic invertebrates and microbes at low‐µg/L to mg/L levels but also have therapeutic potential at low doses; environmental non‐target risks remain under‐characterised	Ale et al. (2018); Bulfon et al. (2013); Čaloudová et al. (2021); Malhotra et al. (2020); Roy and Nath (2022)

## 7 Prospects for the Future and Emerging Trends

### 7.1 Green Synthesis and Sustainable Production Methods

The production of NISs for aquaculture becomes environmentally sustainable through green chemistry and green synthesis methods that focus on using harmless reagents and minimising waste while optimising energy use from start to finish. The development of NIS manufacturing at scale depends on three fundamental principles: solvent‐free or low‐toxicity solvent processes and renewable and biogenic reagents and reaction design for safer product production (Dhingra et al. 2010). Plant extracts and enzyme systems and microorganisms in biological synthesis create safer aquatic environments through their ability to produce biocompatible coatings under mild conditions that generate fewer dangerous by‐products (Dezfooli et al. 2019). The method provides better biocompatibility and decreased metal ion release and lower energy requirements but faces difficulties in mass production and maintaining product consistency and requires full testing to maintain its stable immune system activation properties (Dezfooli et al. 2019). The application of natural biomolecules in green synthesis methods results in better NIS biocompatibility because it generates minimal long‐lasting environmental waste and reduced environmental dangers (Babu et al. 2024). The integration of life‐cycle thinking with safe‐by‐design frameworks allows for trade‐off assessment and supports product development through regulatory frameworks (Hong et al. 2023). Importantly, green synthesis should be viewed not only as an environmentally friendly production strategy but also as a way to improve the biological compatibility of NPs within the fish body. Plant extracts, polysaccharides, proteins, enzymes and microbial metabolites can act as natural reducing and capping agents, forming biogenic surface coatings on AgNPs, ZnONPs and other metal‐based NPs. These coatings may reduce metal ion leakage, limit oxidative stress, improve colloidal stability in gastrointestinal fluids, and enhance cellular tolerance after uptake. Therefore, green‐synthesised NPs may provide safer interactions with fish tissues while maintaining immunostimulatory activity, making this approach particularly relevant for sustainable aquaculture applications.

### 7.2 Integration With Advanced Aquaculture Systems

NISs achieve their best integration with modern aquaculture through the combination of nano‐delivery systems with system‐aware administration methods and compatibility testing and monitoring protocols. First, nano‐formulations, including encapsulated β‐glucans, plant‐derived polysaccharides and peptide adjuvants, can protect bioactive compounds from degradation and enable targeted delivery to gut and mucosal tissues, enhancing immunostimulatory efficacy and reducing dose frequency (Jazayeri et al. 2021; Khanjani et al. 2022; Meena et al. 2013). The combination of recirculating and biosecure systems with nanomaterial‐based water treatment and pathogen control systems enables the reduction of AMR while minimising antibiotic usage (Nasr‐Eldahan et al. 2021; Vijayaram et al. 2023). The design of systems for compatibility demands that particles have dimensions and surface chemistry and release patterns that correspond to both tank materials and target species mucosal sites (gills, skin and gut) to achieve low sorption and toxicity while achieving high uptake (Jazayeri et al. 2021; Meena et al. 2013). The fourth advantage of advanced systems with smart feeders and in‐tank and immersion delivery and real‐time sensors allows NISs dosing based on health indicators and environmental cues, which results in better performance and reduced waste (Iqbal et al. 2024; X. Yang et al. 2020). The implementation of nanotechnology requires evidence from nanotoxicology and immunostimulation studies across species to meet safety and regulatory standards for sustainable use (Nasr‐Eldahan et al. 2021; Vijayaram et al. 2023).

### 7.3 Precision Aquaculture and Smart Delivery Systems

The delivery of NISs requires real‐time health data from Internet of things (IoT) sensors to feed artificial intelligence systems that track fish health status. The implementation of continuous sensor networks for water quality monitoring and temperature and dissolved oxygen measurement and fish activity tracking, together with health proxies such as immunological markers and stress hormones and behavioural indicators, allows for adaptive dosing. AI models enable pathogen risk prediction, which helps determine the best application time and dosage and delivery method between feed and immersion and in‐tank delivery to achieve maximum results and reduce waste (Barton 2002; Ruyra et al. 2013; X. Yang et al. 2020). Smart nanocarriers contain mechanisms that detect physiological signals, including pH changes, redox states and ROS levels, to deliver their payload at specific times when health signals are present (Kaushik et al. 2022; Lavrador et al. 2018; Saravanakumar et al. 2016). The combination of IoT‐enabled actuators, including smart feeders and dosing pumps, with edge/cloud AI systems enables fast modifications to environmental and health indicators, which minimises non‐target exposure while delivering precise amounts to target tissues such as gut/mucosal tissues and gill surfaces through tailored carrier systems (Alam 2023; He et al. 2023; X. Yang et al. 2020). The implementation of stress biomarker monitoring and species‐specific tolerance within control loops enables both fish welfare and safety maintenance and sustainable operation compliance with regulations (Davis 2006; Pankhurst 2011).

### 7.4 Commercialisation Challenges and Market Prospects

The commercial adoption of NISs in aquaculture faces three main obstacles that stem from scientific challenges, economic considerations and regulatory requirements. The scientific community faces three major barriers to confidence and adoption because of insufficient large‐scale field testing and inconsistent product formulations and unclear efficacy results across different species (Song et al. 2017; Woo 2025). The commercialisation and scale‐up of bio‐based products encounter major hurdles because of regulatory barriers and high expenses, which create difficulties in resolving biosafety problems and environmental issues and obtaining reimbursement approval (Davies et al. 2014, 2017). Market fragmentation combined with insufficient evidence about these products' superior performance to traditional immunostimulants leads to high start‐up expenses and production costs and makes investment returns unpredictable (Davies et al. 2014; Kreiss et al. 2020). This unpredictability is further compounded by high manufacturing costs associated with transitioning from milligram‐scale laboratory batches to industrial‐scale production pipelines, which require substantial capital investment for advanced nano‐formulation infrastructure to achieve commercial scalability. The market shows promise because customers increasingly want green disease management solutions and vaccine integration methods, and research studies and technology adoption data indicate positive growth when government backing continues to support the industry (Kreiss et al. 2020). The implementation of modular production systems for manufacturing cost‐effectiveness involves scaling up operations while maintaining quality standards through field testing and strategic partnerships for technology sharing and regulatory compliance to achieve large‐scale production (Ardito et al. 2025; Mladineo et al. 2016; H. Yang et al. 2017). A critical component of this scalability involves successful technology transfer to commercial aquafeed production systems, ensuring that structurally sensitive nanocarriers can withstand the harsh, high‐temperature and high‐pressure conditions characteristic of industrial twin‐screw extrusion and pelleting processes. The market has expanded because of worldwide aquaculture growth and increasing demand for health solutions that do not use antibiotics (Omolloh 2024; Song et al. 2017). Despite this market potential, the practical application of NISs in aquaculture remains constrained by several translational bottlenecks. Regulatory approval poses a significant challenge, requiring product‐specific, longitudinal evidence on NP characterisation, dose–response relationships, acute and chronic toxicity and precise target tissue clearance kinetics. Furthermore, defining standardised withdrawal periods to guarantee zero residual hazards in edible fish fillets at harvest, alongside tracking sediment persistence and long‐term impacts on non‐target aquatic organisms, remains a strict prerequisite for global regulatory clearance. Economic feasibility should also be demonstrated under commercial farming conditions rather than inferred only from laboratory efficacy because production cost, feed‐processing compatibility, storage stability, dose frequency, palatability and return on investment will determine adoption by producers. To establish true economic feasibility under real‐world conditions, rigourous bioeconomic models must demonstrate that the added cost of nano‐enhanced diets is fully offset by measurable reductions in FCRs and avoided disease‐related biomass losses. From a scalability perspective, biodegradable polymeric and lipid‐based systems appear closer to practical use because they are more compatible with oral feed delivery and generally present lower persistence concerns than metallic or carbon‐based nanomaterials. However, even these platforms require standardised manufacturing, batch‐to‐batch quality control, long‐term feeding trials and farm‐scale validation across species, water chemistry, production systems and pathogen challenge scenarios. Therefore, future application should follow a staged validation pathway that moves from laboratory dose optimisation to pilot‐scale trials and then to full production‐cycle field studies integrating efficacy, safety, environmental monitoring and cost–benefit analysis. Ultimately, the integration of AI‐ and IoT‐enabled smart farming systems with NIS delivery could transform aquaculture health management by enabling real‐time stress detection, precision dosing and automated feed‐based administration before disease outbreaks occur.

### 7.5 Emerging New Technologies in Nano‐Immunostimulation

To fully realise the potential of NISs, their integration with cutting‐edge technologies is essential. Recent innovations are shifting towards smart nanocarriers equipped with stimuli‐responsive properties (e.g., pH‐ or enzyme‐triggered release), ensuring that the immunostimulant is released only during active pathogen invasion. In addition, the emergence of RNA‐based nanovaccines and CRISPR/Cas‐edited nano‐delivery systems offers unprecedented precision in targeting specific immune pathways in fish. Beyond the biological level, the fusion of nanotechnology with AI and the IoT, often termed ‘precision aquaculture’, enables real‐time monitoring of fish health biomarkers in pond water. These AI‐driven sensor networks can autonomously trigger the release of nano‐encapsulated prophylactic feeds precisely when stress or early disease signals are detected, drastically reducing waste and maximising therapeutic efficacy. Ultimately, the advancement of NISs aligns with broader technological frontiers in sustainable aquaculture, where the integration of advanced cellular scaffolding, field‐ready encapsulated synbiotics, AI‐driven precision monitoring, phytogenic detoxification mechanisms and multi‐omics‐guided aquafeed formulations synergistically drive the industry towards a resilient, One‐Health‐oriented future (Öz and Üstüner 2026a, 2026c, 2026d, 2026e; Öz, Üstüner, Çifci, et al. 2026).

## 8 Conclusions

This review highlights the growing potential of nanotechnology‐based immunostimulants as a transformative innovation for sustainable aquaculture. Evidence compiled across experimental studies indicates that chitosan, alginate and PLGA‐based nano‐formulations can improve immune biomarkers, bioavailability, mucosal delivery and disease‐resistance outcomes in several aquaculture species. However, the magnitude of protection varies substantially according to fish species, pathogen challenge model, NP composition, dose, administration route and rearing conditions. Therefore, these systems should be regarded as promising but context‐dependent tools rather than universally effective alternatives.

The collective findings suggest that NISs provide an effective strategy to address critical challenges in modern aquaculture, including disease management, AMR and environmental sustainability. Their integration within fish health management frameworks aligns with the One Health approach by reducing antibiotic dependency and preventing AMR gene dissemination across aquatic ecosystems.

Furthermore, NISs present a scientifically sound and environmentally responsible pathway for expanding food production. By ensuring both biological efficiency and ecological safety, they can help the aquaculture industry achieve sustainable intensification without compromising environmental integrity or food safety standards. However, a critical gap remains in the translation of these technologies from laboratory scales to commercial fish farms. The economic viability of large‐scale NP production and the long‐term ecological impact of their accumulation in sediments are not yet fully resolved. Therefore, future research must move beyond efficacy trials to address these scalability and safety challenges.

Future progress should prioritise green synthesis protocols, toxicological standardisation and regulatory harmonisation while fostering interdisciplinary collaboration among nanotechnologists, immunologists and aquaculture specialists. With such coordinated efforts, NISs may become a cornerstone of antibiotic‐free, welfare‐centred and climate‐resilient aquaculture in the decades ahead.

## Author Contributions

**Mustafa Öz**: conceptualisation, visualisation, writing – review and editing, writing – original draft, supervision, resources, software. **Enes Üstüner**: conceptualisation, validation, software, resources, writing – original draft, writing – review and editing.

## Funding

The authors have nothing to report.

## Ethics Statement

The authors have nothing to report.

## Conflicts of Interest

The authors declare no conflicts of interest.

## Data Availability

The authors have nothing to report.
